# Encoding of continuous perceptual choices in human early visual cortex

**DOI:** 10.3389/fnhum.2023.1277539

**Published:** 2023-11-13

**Authors:** Riccardo Barbieri, Felix M. Töpfer, Joram Soch, Carsten Bogler, Henning Sprekeler, John-Dylan Haynes

**Affiliations:** ^1^Bernstein Center for Computational Neuroscience and Berlin Center for Advanced Neuroimaging, Department of Neurology, Charité – Universitätsmedizin Berlin, Corporate Member of Freie Universität Berlin, Humboldt-Universität zu Berlin and Berlin Institute of Health (BIH), Berlin, Germany; ^2^German Center for Neurodegenerative Diseases, Göttingen, Germany; ^3^Department for Electrical Engineering and Computer Science, Technische Universität Berlin, Berlin, Germany; ^4^Berlin School of Mind and Brain and Institute of Psychology, Humboldt-Universität zu Berlin, Berlin, Germany

**Keywords:** continuous decision making, functional magnetic resonance imaging, encoding model, Gaussian process regression, early visual cortex

## Abstract

**Introduction:**

Research on the neural mechanisms of perceptual decision-making has typically focused on simple categorical choices, say between two alternative motion directions. Studies on such discrete alternatives have often suggested that choices are encoded either in a motor-based or in an abstract, categorical format in regions beyond sensory cortex.

**Methods:**

In this study, we used motion stimuli that could vary anywhere between 0° and 360° to assess how the brain encodes choices for features that span the full sensory continuum. We employed a combination of neuroimaging and encoding models based on Gaussian process regression to assess how either stimuli or choices were encoded in brain responses.

**Results:**

We found that single-voxel tuning patterns could be used to reconstruct the trial-by-trial physical direction of motion as well as the participants’ continuous choices. Importantly, these continuous choice signals were primarily observed in early visual areas. The tuning properties in this region generalized between choice encoding and stimulus encoding, even for reports that reflected pure guessing.

**Discussion:**

We found only little information related to the decision outcome in regions beyond visual cortex, such as parietal cortex, possibly because our task did not involve differential motor preparation. This could suggest that decisions for continuous stimuli take can place already in sensory brain regions, potentially using similar mechanisms to the sensory recruitment in visual working memory.

## 1. Introduction

The brain mechanisms of perceptual decisions involve several sequential steps (Gold and Shadlen, 2007): first, information about an external stimulus is encoded in sensory brain regions (e.g., whether an object is moving leftward, or rightward). Then, the sensory evidence supporting the different potential states of the world is gathered in a decision variable, which typically also integrates information over time (but see Uchida et al., 2006; Gold and Shadlen, 2007). Finally, once enough evidence is collected in favor of a certain hypothesis, a decision is made, and the observer takes an action to indicate their choice.

Several stages of this processes have been characterized in detail, both in humans and in monkeys (Gold and Shadlen, 2007; Heekeren et al., 2008; Mulder et al., 2014; Forstmann et al., 2016; Hanks and Summerfield, 2017). In monkeys, sensory neurons encode stimulus-related information, such as the physical direction of movement of dots in the visual field (Shadlen et al., 1996). Regions of parietal and frontal cortex encode the gradual accumulation of choice-related, categorical evidence in monkeys (Gold and Shadlen, 2007) and in humans (Siegel et al., 2011; Mulder et al., 2014; Wilming et al., 2020). Neuroimaging signals also reflect levels of evidence (Heekeren et al., 2004; Forstmann et al., 2016) and strategic adjustments in decisions (Mulder et al., 2014). As for the outcome of the decision, many human neuroimaging studies have identified choice-related brain signals in several areas including parietal cortex (Tosoni et al., 2008, 2014; Liu and Pleskac, 2011; Hebart et al., 2012, 2016; Levine and Schwarzbach, 2017), insular cortex (Ho et al., 2009; Liu and Pleskac, 2011) and prefrontal cortex (Heekeren et al., 2004, 2006; Filimon et al., 2013; Hebart et al., 2016). Interestingly, studies have shown that activity already in sensory areas is influenced by the behavioral choice, especially in ambiguous stimulus conditions (Ress and Heeger, 2003; Serences and Boynton, 2007; Sousa et al., 2021).

Due to this variety of decision signals, the representational space in which decision outcomes are encoded has remained somewhat unclear, with some suggesting a motor-based “intentional” frame of reference (Shadlen et al., 2008; Tosoni et al., 2014) whereas others have shown that decision signals can be dissociated from motor plans in both monkeys and humans (Bennur and Gold, 2011; Hebart et al., 2012; Filimon et al., 2013; Park et al., 2014; Brincat et al., 2018, but see discussion below).

Importantly, there is an issue that has only received little attention: studies of perceptual decision making have typically employed few discrete alternative stimulus features, whereas perception of most sensory features is inherently continuous (Levinson and Sekuler, 1976; Albright, 1984; Movshon and Newsome, 1996; Prinzmetal et al., 1998; Nichols and Newsome, 2002; van Bergen et al., 2015). For example, most studies that use perceptual judgements of coherent motion focus on few alternative motion directions in each trial and require categorical responses (Gold and Shadlen, 2007; Churchland et al., 2008; Huk and Meister, 2012; Hanks and Summerfield, 2017). In these tasks a small set of possible motion directions (e.g., motion left or motion right) has to be mapped onto a small set of predetermined motor responses (e.g., pressing the left or the right button or making a saccade to a left or right target). In such cases, participants might encode their choices in a motor frame of reference or in a lower-dimensional categorical form (“left” vs. “right”). In contrast, choices could also be encoded in some kind of continuous perceptual space (Beck et al., 2008; van Bergen et al., 2015; Smith, 2016; Ratcliff, 2018). Note that even if a paradigm allows to dissociate choices from motor plans by the use of trial-wise varying stimulus-response mappings (Bennur and Gold, 2011; Hebart et al., 2012), the encoding of choices still occurs in the form of such stimulus-response-mappings and thus uses discrete, lower-dimensional representations. Alternatively, however, choices could be encoded on a full 360° continuum.

Here we assessed the encoding of continuous perceptual choices using a combination of fMRI and voxel-wise fMRI encoding models (Nevado et al., 2004; Thirion et al., 2006; Dumoulin and Wandell, 2008; Kay et al., 2008; Brouwer and Heeger, 2009; Naselaris et al., 2011; Haynes, 2015; see “2. Materials and methods” for full details). As stimuli we used random dot kinematograms (RDKs) as in many studies of perceptual decision making (Newsome and Paré, 1988). These stimuli consist of an array of dots moving in various directions like a detuned TV-set. By modifying the proportion of dots that coherently move in a single target direction (signal) among others moving in random directions (noise), it is possible to assess perceptual decisions under varying levels of sensory information.

**FIGURE 1:**
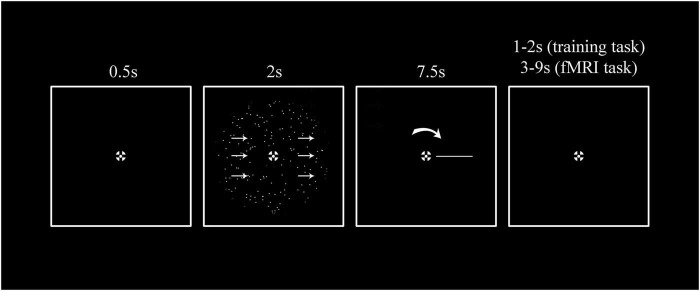
A single trial stimulus sequence. Participants were instructed to fixate the center of a bullseye for the entire duration of the experiment. 0.5 s after trial onset, they were presented with 2 s of random dot motion stimulus (RDK) displayed with a different direction of motion and coherence level for every trial. The direction of motion was continuously distributed and spanned between 0 and 360°. For the training task participants were presented with five different coherence levels (0, 12.5, 25, 50, and 100%). For the fMRI task, instead, only three coherence levels were presented (0%, an intermediate coherence level individually estimated during the training phase, and 100%). Based on previous experiments (Töpfer et al., 2022) we used a report method that involved a rotating perceptual comparison stimulus and thus was given in a perceptual (as opposed to a motor) frame of reference. This has the advantage of increasing accuracy and minimizing bias (Töpfer et al., 2022). Specifically, participants were instructed to report the net motion direction by pressing the response button when a self-moving rotating bar on the screen matched the direction of motion they perceived. After the report was given the bar continued its cycle so that the presentation duration was always 7.5 s. Inter-trial intervals (ITIs) for the training task were 1 or 2 s, for the fMRI task they were between 3, 5, 7, and 9 s, exponentially distributed so that shorter ITIs happened more frequently than longer ones.

For our feature-continuous motion stimuli the directions were drawn from a uniform distribution between 0 and 360°. Participants reported their judgements by pressing a button when a rotating sensory comparison stimulus matched their choice (Figure 1). In previous work we found that reports like this that used a sensory reference stimulus, instead of e.g., the movement of a track ball, had the highest accuracy for continuous judgements (Töpfer et al., 2022). It also allows to decouple choice-related signals from specific motor preparation. We measured trial-by-trial brain activity under three different coherence levels: 0%, intermediate and 100% coherence. Note that at 0% coherence there is no physical evidence regarding the stimulus direction and participants are purely guessing. This condition is of particular importance because it allows to study choices independent of physical stimulus information, and it will be the primary focus of our analyses.

Our main research question is whether cortical brain areas encode choices for motion directions in a continuous perceptual space. More specifically, we hypothesized that voxels in visual areas are tuned to the stimulus-graded motion directions and to perceptual choices about motion direction. We predicted that this information could be used to reconstruct the trial-by-trial stimulus and the reported motion direction, respectively. We expected the sensory and choice-related information encoded by these voxels to decrease as a function of coherence, thus resulting in lower levels of reconstruction accuracy at lower coherence levels. While reconstruction of sensory information should result in chance-level performance in the 0% coherence condition, we expected visual areas to carry sufficient information for performing choice reconstruction. We additionally wanted to test whether information encoded in MT+ could be used to reconstruct the stimuli and the corresponding reports. Previous studies have shown the importance of MT+ in motion perception (Newsome and Paré, 1988; Britten et al., 1996; Shadlen et al., 1996; Rees et al., 2000; Braddick et al., 2001), but there have been diverging reports on whether motion direction can be decoded from this area using fMRI (Kamitani and Tong, 2006; Serences and Boynton, 2007; Beckett et al., 2012; Hebart et al., 2012; Wang et al., 2014). Finally, we wanted to assess, if sensory and choice-related information is encoded in parietal cortex, a part of the brain that is suggested to code for a mixture of task-related properties including the outcome of perceptual choices (Bennur and Gold, 2011; Rigotti et al., 2013; Fusi et al., 2016; Brincat et al., 2018).

## 2. Materials and methods

### 2.1. Preregistration

The hypotheses, methods and analyses employed in this study were preregistered at https://osf.io/e2bvn before analyzing the data. Any additional exploratory analyses that go beyond what is specified in the preregistration are explicitly marked as such below.

### 2.2. Data and code availability

The datasets generated for this study can be found in OSF https://osf.io/vcmdg/, whereas the code is available on Github https://github.com/RiccardoBarb/GPR_fMRI.

### 2.3. Participants and exclusion criteria

We recruited participants from several sources. Some were contacted using an internal mailing list consisting of people who previously participated in fMRI experiments in our lab. Others were recruited from Facebook groups for English-speaking jobs in Berlin and Berlin university students. All of the participants gave written informed consent, were paid 7€/h for the behavioral training session and 10€/h for the fMRI sessions. Those who completed all of the experimental sessions (1 training + 2 fMRI) received an additional bonus of 50€. The research protocol was conducted in accordance with the Declaration of Helsinki and approved by the local psychological ethics committee.

We selected healthy right-handed subjects with no history of neurological or psychiatric diseases. Furthermore, following our previous studies (Töpfer et al., 2022), we decided to exclude participants prior to scanning on the basis of their performance in a behavioral training session.

A participant was excluded if they were not sufficiently precise in the indication of motion direction, which was defined as the 95% percentile of the △*x* distribution (see below, eq.1) in the full coherence condition exceeding a cutoff of 36.5°.

We also excluded participants that were not able to correctly perceive the stimulus in another, more systematic way. We had previously observed that some subjects frequently mistake a motion direction with its 180-degree opposite, a phenomenon that would be mistaken for a guess in most conventional categorical motion judgment tasks. For this reason, we employed a von Mises mixture model (vMMM) to quantify the frequency of reports of opposite direction (ROOD; Töpfer et al., 2022). ROOD rates exceeding 5% at full coherence led to the exclusion of a participant.

We initially collected behavioral training data from 41 subjects. Of these, 13 were excluded after the training phase prior to scanning following the abovementioned exclusion criteria. Three participants did not complete the MRI sessions for technical reasons and were excluded from the subsequent analyses; thus, 25 participants completed the fMRI sessions. One participant with low behavioral performance in the training session was accidentally included in scanning and was subsequently removed leading to a total of 24 participants who successfully concluded the experiment according to our pre-defined criteria (9 females; age range: 18–34; mean age: 25.6; SD: 4.6). We used the data of one participant to develop and check our Gaussian process regression (GPR) pipeline. In order to avoid any circularity or overfitting this participant was not included in the final analyses, which led to a total sample size of 23 subjects considered for all the statistical analyses.

### 2.4. Visual stimuli

#### 2.4.1. General features

The random dot kinematograms (RDKs—Figure 1) consisted of white dots moving inside a circular aperture on a black background. The aperture was centered on the screen and had an inner diameter of 2.5 dva and an outer diameter of 15 dva. Alpha blending was applied at the borders to avoid sharp contrast boundaries. For this, the luminance of the dots was progressively reduced before they wrapped around the other side of the annulus. The dot size was 0.1 dva (Braddick, 1973). The motion speed of the dots was 6°/s (van de Grind et al., 1983; Geisler, 1999) and the dot density was 1.6 dots/dva2 (Downing and Movshon, 1989), leading to a total of 275 dots. A white bullseye fixation target (Thaler et al., 2013) was placed in the central aperture spanning 0.25 dva. The mean luminance measured on the white center of the bullseye was 17.5 cd/m^2^. The mean luminance measured on the black background was 0.206 cd/m^2^.

#### 2.4.2. Directions of motion

To pseudo-randomize the directions across trials, while maintaining the continuous nature of the task, we separated the stimuli into 8 hidden randomization bins. Each bin divided the stimulus space into equal portions of 45°. The bin edges were set at 337.5, 22.5, 67.5, 112.5, 157.5, 202.5, 247.5, 292.5° (0° pointing up, 90° pointing right). Within a bin, the direction of motion was uniformly randomly distributed. In this way we made sure that the motion direction varied continuously across trials, while respecting some experimental constraints. We used an equal amount of trials for each subject in each directional bin, the same bin did not occur more than twice in a row and the same coherence level was not presented more than three times in a row. Please note that in the 0% coherence condition, no net motion direction is present. Directional labels are still assigned following the same randomization scheme adopted for the other coherence conditions, but there is no relationship between the labels and the underlying motion of the stimulus.

### 2.5. Training session

#### 2.5.1. Experimental setup

In order to train participants on the task lying-down in supine position (as during MR-scanning), we used a custom-built mock scanner. The training phase of the experiment took place in a dimly illuminated room (mean background luminance as measured on a white wall: 0.0998 cd/m^2^), where participants were lying in this mock scanner. They placed their head on a pillow and viewed a DELL LCD monitor 35 cm wide through a reflecting mirror. The monitor was set with 60 Hz refresh rate and a resolution of 1,024 × 768 pixels. The stimuli were generated and presented using MATLAB R2016a (The MathWorks Inc., Natick, MA, United States) and Psychtoolbox 3 (Brainard, 1997; Kleiner et al., 2007). For behavioral training, participants had their right hands on a standard computer keyboard placed on their hips.

#### 2.5.2. Training task

For the training session, each trial started with the presentation of a fixation bullseye which remained present throughout the whole duration of the trial (see Figure 1). Participants were instructed to fixate the center of the bullseye for the entire duration of the trial. After 0.5 s, participants were presented for 2 s with a random dot motion stimulus (RDK) that had a different direction of motion and coherence level for every trial. The direction of motion was continuously distributed between 0 and 360° and its order of presentation was subject to constraints (see above). There were five different coherence levels in the training phase: 0, 12.5, 25, 50, 100%. After termination of the stimulus, participants gave a judgment of motion direction using a report that employs a perceptual frame of reference with a visual comparsion stimulus (see Figure 1). In a previous study (Töpfer et al., 2022) we observed that this method of responding avoided systematic biases that are observed when using continuous reports involving trackballs that employ a motor frame of reference. Specifically, after offset of the motion stimulus a self-moving rotating bar was presented inside the aperture. Participants were asked to indicate the net motion direction of the dots by pressing the response button as soon as they believed the bar on the screen to match the direction of motion they perceived. The bar pointed from the center of the aperture to the outer border of the stimulus (like the arm of a clock), starting from a random position in every trial. The bar was 7.5 dva in length. It was randomly chosen to rotate clockwise or counterclockwise around the central fixation at a speed of 0.2 cycles/second, and it kept rotating after the response was given so that the total rotation time was always 7.5 s. Participants were instructed to always respond as precisely as they could, even if they were unsure. On some trials (catch trials), a portion of the rotating bar changed contrast after the response indication. Participants were instructed to press the response button as fast as possible when they detected the contrast change. The purpose of such trials was to make sure that participants were paying attention to the bar rotation throughout the entire duration of the response period (even after they indicated their response), while maintaining fixation. In the training task, participants’ responses were followed by a uniform inter-trial interval (ITI) of 1 or 2 s, after which a new trial started. Subjects performed 9 blocks of 40 trials during the training phase. An additional block was used to estimate the exact level of coherence that yielded intermediate performance using the QUEST staircase method (Watson and Pelli, 1983).

### 2.6. Experimental session

#### 2.6.1. fMRI experimental task

Participants who completed the training and matched our performance requirements (see section “2.3. Participants and exclusion criteria”) were scheduled for 2 different MRI sessions on 2 different days. Participants performed a total of 10 experimental runs (5 runs in each session). The structure of the training and the experimental tasks were essentially the same, except for the inter-trial interval (ITI), which was chosen between 3, 5, 7, and 9 s, where ITI frequency was exponentially distributed (shorter ITIs were more likely than longer ones). Moreover, in the scanning sessions, the RDK was presented with three coherence levels instead of five: 0%, an intermediate coherence level estimated for each subject from the training phase data (mean coherence level: 19.47%; SD: 5.3%), and 100%. Each coherence level was presented 16 times in each run which resulted in 48 trials per run for a total of 10 runs. Participants were required to maintain fixation for the entire duration of the trial. Their eye position was monitored with an MRI compatible EyeLink 1000+ eyetracking system.

#### 2.6.2. fMRI localizer task

After the experimental runs, participants performed 2 runs of an MT-localizer task on each day, for a total of 4 runs on both days. In the localizer, their task was to passively view the presented stimulus while maintaining fixation. After 0.5 s of bullseye, they viewed an 8 s RDK at 100% coherence with random directions changing at a frequency of 2 Hz, followed by 8 s RDK at 0% coherence. The ITI was implemented in the same way as in the main fMRI task. Participants were instructed to fixate the center of a bullseye for the entire block. The total number of blocks for each run was 32 (16 coherent, and 16 incoherent). This particular design has been proven effective in eliciting a strong BOLD signal in motion-sensitive visual areas (Braddick et al., 2001).

#### 2.6.3. MRI data acquisition

Functional MRI data were acquired on a 3T Siemens Prisma scanner (Siemens, Erlangen, Germany) equipped with a 64-channel head-coil, using a T2-weighted multi-band accelerated EPI sequence (from the Human Connectome Project–HCP) with a multiband factor of 8. The fMRI runs (TR = 800 ms, TE = 37 ms, flip angle = 52°, voxel size = 2 mm × 2 mm isotropic, 72 slices, 1.9 mm inter-slice gap) were preceded by a high-resolution T1-weighted MPRAGE structural scan (208 sagittal slices, TR = 2,400 ms, TE = 2.22 ms, flip angle = 8°, voxel size = 0.8 mm^2^ isotropic, FOV = 256 mm). The MRI sessions took place over the course of 2 days. Each day comprised 5 experimental runs (805 whole-brain volumes per run) and 2 functional localizer runs (480 whole-brain volumes per run). The first 4 TRs were discarded to allow for magnetic saturation effects.

#### 2.6.4. Eye-tracking data acquisition

Horizontal and vertical gaze position as well as the area of the pupil, were recorded from each subject’s dominant eye in the MRI scanner using an EyeLink 1000+ (SR-Research, sampling rate 1,000 Hz) with long distance mount. Calibration took place before the experiment once at the beginning of every session.

### 2.7. Data analysis

#### 2.7.1. Behavioral measures of performance accuracy

The absolute trial-by-trial circular response deviation from the target direction was used as a primary measure of performance accuracy:


(1) $$\left|△\,x\right|=\left|\theta_{s}-\theta_{r}\right|$$

where θ_*s*_ is the stimulus direction and θ_*r*_ is the reported direction. Furthermore, as in previous studies we rescaled the absolute deviation in the range 0–100% (feature-continuous accuracy, FCA—see also Pilly and Seitz, 2009):


(2) $$F\,C\,A=\frac{180^{{}^{\circ}}-\left|△\,x\right|}{180^{{}^{\circ}}}\times 100$$

Here chance performance, i.e., randomly guessing the continuous direction, corresponds to an average FCA of 50% (or |△*x*|=90°) and perfect performance, i.e., identically matching the presented direction, corresponds to an FCA of 100% (or |△*x*|=0°). This approach has the advantage of providing a trial-by-trial measure of performance, which is interpretable at all coherence levels (including 0% coherence) and facilitates the comparison with more conventional 2-choice accuracies. An alternative to this approach would consist in fitting a mixture model to obtain an estimate of detection and guessing (Zhang and Luck, 2008; Bae and Luck, 2019; Töpfer et al., 2022). However, at 0% coherence, when there is no motion direction information available and subjects are purely guessing, the model fit would provide uninterpretable estimates for the detection parameter (Töpfer et al., 2022).

#### 2.7.2. Regions of interest (ROIs) definition

The fMRI data from the localizer runs were first spatially realigned, coregistered to individual anatomical images (Glasser et al., 2013) and spatially smoothed using a Gaussian kernel with an FWHM of 6 mm. For each subject, we then modeled the activity during the localizer in each voxel using a general linear model implemented in SPM12. For each of the 4 runs, we included 1 regressor for coherent motion and 1 for incoherent motion, as well as 6 regressors of no interest to account for participants’ head movement. We then performed two univariate analyses: the first assessed in which voxels the BOLD signal was stronger during coherent compared to incoherent motion (for definition of MT+, see below). The second assessed where both coherent and incoherent motion activated voxels above baseline (for early visual and parietal brain regions, see below). The statistical maps obtained with this contrast were corrected for multiple comparison and thresholded at *p* < 0.05 (FWE). Regions of interest (ROIs) can be seen on the left part of Figure 6.

##### 2.7.2.1. Early visual cortex (EVC)

Early visual cortex was defined based on a combination of a spatially normalized functional mask of motion-related activity and an anatomical mask defined by the union of V1, V2 and V3. Unlike MT+ masks, the functional EVC mask was defined at the group level because we did not expect significant inter-individual differences in the activation maps elicited by our contrast. Specifically, the functional activation elicited by both localizer stimuli (coherent and incoherent against baseline) constituted a large cluster (*p* < 0.05, FWE) at the occipital pole. Please note that this voxel selection is independent of motion coding information. For the anatomical mask we employed the SPM Anatomy Toolbox (Eickhoff et al., 2005) to define an anatomical mask spanning the occipital areas hOC1, hOC2 (Amunts et al., 2000), hOC3d (Rottschy et al., 2007) and hOC3v (Kujovic et al., 2013). Our early visual ROI is defined as the intersection between the functionally and the anatomically defined masks.

##### 2.7.2.2. Area MT+

The motion complex MT+ was identified as the set of voxels activated more to the coherent than the incoherent localizer stimuli within a sphere (*r* = 10 mm) located in the center of the significant clusters lateral to the parietal-occipital sulcus bilaterally (see Figure 6 for the MT+ ROI of an example subject).

##### 2.7.2.3. Parietal areas

We used the SPM Anatomy Toolbox (Eickhoff et al., 2005) to further select voxels from three different subregions of parietal cortex that have previously been reported as informative about behavioral choices in similar perceptual decision-making experiments (Hebart et al., 2012, 2016; Bode et al., 2013): superior parietal cortex (SPC–areas 5L, 5M, 5Ci, 7A, 7PC, 7M, 7P; Scheperjans et al., 2008), inferior parietal cortex (IPC–areas PFop, PFt, PF, PFm, PFFcm, PGa, PGp; Caspers et al., 2006) and intraparietal sulcus (IPS–areas hlP 1-3; Choi et al., 2006; Scheperjans et al., 2008).

#### 2.7.3. Selection of trials based on eye-tracking

In order to avoid potential eye-movement confounds in our main fMRI analysis we checked that participants maintained fixation using the eye-tracker data. For this, in a first step we used a preprocessing pipeline adapted from Urai et al. (2017). Missing data and blinks were not interpolated for fixation control. The standard deviation of the gaze position was estimated for every run and every subject. We obtained the probability density function of the distribution of all standard deviations of eye positions, collapsed across all subjects and runs, using a kernel density estimation. A noise threshold was defined by estimating the inverse of the cumulative density at the probability of 0.9. This is equivalent to excluding the 10% of the noisiest runs based on the fixation analysis. Furthermore, trials where subjects exceed a deviation threshold of 2 dva for more than 200 ms during the stimulation period were rejected from the analysis of the neuroimaging data. The eye fixation control resulted in the exclusion of 295 trials, or an average of 2.3% of trials for each participant (mean number of trials: 11.13; SD: 14.51). Together with trials in which participants did not report their perceived direction, we excluded an average of 2.5% trials per subject (mean number of trials: 12.17; SD: 14.42). A repeated-measures ANOVA revealed no significant difference in the amount of excluded trials across coherence levels [F (2,44) = 1.296, *p* = 0.284].

#### 2.7.4. Statistical analyses of behavioral performance

In order to evaluate the effect of coherence on behavioral performance, we performed a repeated-measure ANOVA on performance accuracy (FCA—see eq. 2), with coherence level as within-subject factor. The test was performed with JASP.

#### 2.7.5. fMRI data analysis

The fMRI data analysis of the main experimental task was performed in MATLAB using SPM 12, the GPML toolbox (Rasmussen and Nickisch, 2010) and custom functions. Before the analysis, data were motion corrected and coregistered to anatomical images. After this preprocessing, the analysis proceeded in four steps: (1) trial-wise and voxel-wise GLM; (2) voxel-wise Gaussian process regression (GPR) estimation; (3) searchlight-based stimulus and report reconstruction; (4) group level analyses.

##### 2.7.5.1. Trial-wise GLM

For each participant, we modeled the fMRI signal acquired in each voxel during the 2 s of the stimulus period with a trial-wise GLM (Rissman et al., 2004). Our model consisted of one regressor per trial and 6 head-motion regressors of no interest for each run.

##### 2.7.5.2. Voxel-wise GPR estimation

In a next step we computed what we refer to as full distribution tuning functions (FDTF) for each voxel to assess how the estimated trial-wise responses in that voxel were modulated either by the stimulus direction θ_*s*_ or by the reported direction θ_*r*_. The key idea of the FDTF over and above conventional voxel encoding models is to not only estimate the mean response in a given voxel as a function of direction, but to estimate the entire ensemble of direction-conditional likelihood functions (for details see below). For this we used Gaussian process regression (Rasmussen and Williams, 2005). The trial-wise parameter estimates were entered into two separate cyclic GPR, models, one for the corresponding stimulus motion direction θ_*s*_ and one for the reported direction θ_*r*_. This yielded a feature-continuous model of the entire distribution of fMRI responses for each direction, akin to a voxel-tuning function that includes not only the mean but also the distribution at each direction. The GPR has the advantage that it does not pre-suppose a fixed number of sensory channels per voxel (Brouwer and Heeger, 2009; van Bergen et al., 2015). This procedure was repeated for each coherence level, leading to the estimation of two separate sets of FDTFs for each voxel, three coherence levels and physical vs. reported directions (six estimated models in total).

Consider the *t* = 160 total trials for one coherence level across runs. Then, let ${\hat{\beta }}_{j}$ be the *t*×1 vector of trial-wise parameter estimates in a single voxel *j* and let θ be the corresponding vector of stimulus or reported motion directions.

We assumed that the response amplitude of each voxel *j* during each trial *i* was a function of this trial’s direction of motion θ_*i*_ ∈ Θ=(0,2π) and the voxel-specific kernel parameters *L_j_*, plus the normally distributed noise term ε_*ji*_:


(3) $${\hat{\beta }}_{j\,i}=f\,\left(\theta_{i},L_{j}\right)+\varepsilon_{j\,i};f:\Theta \to \mathbb{R}$$

The function *f* is intentionally specified unconstrained, because it was estimated using voxel-wise GPR of responses ${\hat{\beta }}_{j}$ against directions θ, such that each voxel *j* obtained a unique response profile *f_j_*. The estimation of such voxel-wise response profiles was performed separately for each coherence level. For this we only used trials in which participants maintained fixation, as evaluated by the analysis of the eye-tracking data (see “2.7.3. Selection of trials based on eye-tracking”). In order to avoid overfitting in the subsequent phases of the analysis (i.e., the searchlight reconstruction – see Kriegeskorte et al., 2006), we estimated the response profiles *f_j_* by including the trials from all runs except one, and repeated the procedure until the trials from all runs were used for the model estimation (leave-one-run-out cross-validation scheme). Each iteration was based on a maximum of 9/10×160=144 datapoints (which could be maximally achieved when no data were excluded after fixation control).

##### 2.7.5.3. Searchlight-based stimulus and report reconstruction

Next, we used the estimated tuning functions to reconstruct the direction for a set of independent test trials. We then combined the estimated ${\hat{\beta }}_{j}$ parameters from a group of voxels within a searchlight (*r* = 4 voxels) into the matrix $\hat{\text{B}}$, to predict the stimulus direction θ_*s*_ or the reported direction θ_*r*_ using a run-wise cross-validation procedure. The estimated voxel-wise response profiles *f*_*j*_ = *f*(θ_*i*_,*L*_*j*_) can be used to predict the 1×*v* vector of response amplitudes across voxels *j* in one trial *i*, i.e., one row of $\hat{\text{B}}$:


(4) $${\hat{\beta }}_{j.}=g\,\left(\theta_{i},\hat{L}\right)+\varepsilon_{j.};g:\Theta \to {\mathbb{R}}^{1\times v}$$

as well as the whole *t*×*v* matrix of trial-wise parameter estimates $\hat{\text{B}}$:


(5) $$\hat{\text{B}}=h\,\left(\theta ,\hat{L}\right)+E;h:\Theta^{t\times 1}\to {\mathbb{R}}^{t\times v}$$

with $\hat{L}=\left\{L_{1},\ldots ,L_{v}\right\}$, where *g* and *h* can be written in terms of *f* as:


(6) $$g\,\left(\theta_{i},L\right)=\left[f\,\left(\theta_{i},L_{1}\right),\ldots ,f\,\left(\theta_{i},L_{v}\right)\right];h\,\left(\theta ,L\right)=\left[\begin{array}{c} g\,\left(\theta_{1},L\right)\\ \vdots \\ g\,\left(\theta_{t},L\right) \end{array}\right]$$

Let’s partition the trial-wise parameter matrix $\hat{\text{B}}$ into training data ${\hat{\text{B}}}_{\text{train}}$ and test data ${\hat{\text{B}}}_{\text{test}}$ and let $\hat{L}=\left\{L_{1},\ldots ,L_{v}\right\}$, be the set of estimated kernel parameters obtained from ${\hat{\text{B}}}_{\text{train}}$. Then, the residuals of the GPR model in the voxel-wise GPR equation are:


(7) $$\hat{R}={\hat{\text{B}}}_{\text{train}}-h\,\left(\theta_{t\,r\,a\,i\,n},\hat{L}\right)$$

and estimated covariance is:


(8) $$\hat{\Sigma }=\frac{1}{t}\,{\hat{R}}^{\text{T}}\,\hat{R}$$

In the case that the number of voxels in a searchlight *v* is larger than the number of trials across runs *t*, the matrix is not invertible, such that it has to be regularized. Here, we chose a shrinkage estimator by mixing in a diagonal matrix of sample variances:


(9) $$\hat{\Sigma }=\left(1-r\right)\cdot \frac{1}{t}\,{\hat{R}}^{T}\,\hat{R}+r\cdot d\,i\,a\,g\,\left(\left[{\hat{\sigma }}_{1}^{2},\ldots ,{\hat{\sigma }}_{v}^{2}\right]\right)$$

Where the mixing coefficient *r* was a function of the voxel-to-trial ratio (arctan regularization):


(10) $$r\,\left(v/t\right)=\frac{1}{\pi }\,\left[a\,r\,c\,t\,a\,n\,\left(l\,n\,\frac{v}{t}\right)\right]$$

or logistic regularization:


(11) $$r\,\left(v/t\right)=\frac{1}{[1+exp\left[-ln\frac{v}{t}\right]}$$

such that $r=\frac{1}{2}$ when $\frac{v}{t}=1$ and lim_*x*→0_*r*(*x*) = 0 as well as lim_*x*→∞_*r*(*x*) = 1.

Let’s consider the responses of all voxels in just one trial. According to the GPR model, those single-trial across-voxel responses are distributed as:


(12) $${\hat{\beta }}_{i.}=g\,\left(\theta_{i},L\right)+\varepsilon_{i.},\varepsilon_{i.}∼𝒩\,\left(0,\Sigma \right)$$

Which implies a multivariate normal log-likelihood function.


(13) $$\text{LL}\left(\theta_{i}\right)=logp\left({\hat{\beta }}_{i.}|\theta_{i}\right)=log𝒩\left({\hat{\gamma }}_{i.};g\left(\theta_{i},L\right),\Sigma \right)$$

where 𝓝(*x*;μ,Σ) is the probability density function of the multivariate normal distribution and Σ is the unknown *v*×*v* spatial covariance matrix.

The stimulus or the reported motion direction in a particular trial of the test set can be reconstructed by maximum-likelihood estimation (MLE), i.e., by simply maximizing the out-of-sample likelihood function, given the in-sample parameter estimates $\hat{L}$ and $\hat{\Sigma }$ :


(14) $${\hat{\theta }}_{i}=\underset{\theta \in \Theta }{\text{argmax}}L\,L\,\left(\theta \right)=\underset{\theta \in \Theta }{\text{argmax}}l\,o\,g\,𝒩\,\left({\hat{\text{B}}}_{\text{test}}^{\left(i\right)};g\,\left(\theta ,\hat{L}\right),\hat{\Sigma }\right)$$

where ${\hat{\text{B}}}_{\text{test}}^{\left(i\right)}$ is the *i*-th row of ${\hat{\text{B}}}_{t\,e\,s\,t}$, containing parameter estimates from the *i*-th test trial. To efficiently determine ${\hat{\theta }}_{i}$, we perform a grid-based search, ranging θ from 1° to 360° in steps of 1° and evaluating LL(θ) at the corresponding values. This approach is similar to inverting a set of forward encoding models (Thirion et al., 2006; Brouwer and Heeger, 2009; Naselaris et al., 2011; Haynes, 2015; Sprague et al., 2018; Kriegeskorte and Diedrichsen, 2019).

##### 2.7.5.4. Reconstruction performance evaluation

The outcome of the reconstruction is the matrix of predicted directions $\hat{\theta }$ where each row represents a specific trial and columns are different searchlights.

Following our preregistration protocol, we evaluated the reconstruction performance in terms of feature-continuous accuracy (FCA, eq. 2). For each searchlight and trial, we compared the true (stimulus or report) direction θ_*t*_ and the predicted direction ${\hat{\theta }}_{t}$ in terms of absolute angular deviation, rescaled into the range 0–100%, according to equations (1) and (2). We then computed the averaged FCA across trials. This measure works well with balanced independent variable distributions, such as that of the stimulus motion directions. However, in order to avoid spurious above-chance reconstruction performance in case of an unbalanced distribution of the dependent variable (i.e., during the reconstruction of participants’ reports which are not equally distribution across the directions, especially at lower coherence levels), we computed a balanced version of FCA (BFCA):


(15) $$\text{BFCA}=\frac{1}{2\,\pi }\,\int_{0}^{2\,\pi }\text{FCA}\,\left(\theta ,\hat{\theta }\right)\,\text{d}\,\theta$$

where the integral is calculated using trapezoidal numerical integration across the sorted directions of motion θ and reconstructions $\hat{\theta }$. Note that, in case of balanced labels, the use of FCA or BFCA produce virtually identical results (see Supplementary material for details).

##### 2.7.5.5. ROI analyses

In order to evaluate how the stimulus and the report reconstruction performance were affected by coherence in early visual cortex, MT+ and parietal areas, we evaluated the effect of coherence level on the reconstruction performance for five bilateral ROIs (see “2.7.2. ROIs definition”). Parietal areas encode a variety of task-related variables (Bennur and Gold, 2011; Rigotti et al., 2013; Park et al., 2014; Fusi et al., 2016; Brincat et al., 2018), including perceptual choices in previous studies where decisions only had few alternative options (Liu and Pleskac, 2011; Hebart et al., 2012, 2016). We expected an effect of coherence on report reconstruction in visual areas and MT+ reflecting the increased correlation between stimulus and response for higher coherence levels. To test these hypotheses, we first computed the average reconstruction performance for each label (stimulus and report) in each ROI for each subject. We then submitted the data to five independent repeated-measures ANOVAs (one for each ROI) with coherence level and reconstructed label as repeated measure factors. The statistical tests for the ROI analyses were performed with JASP (JASP Team, 2020).

#### 2.7.6. Additional exploratory analyses

In order to deepen our understanding of the relationship between coherence level and reconstruction performance, and to explore the similarity between the stimulus and report GPR models, we further performed two exploratory analyses. The analyses were performed on an ROI defined by the intersection of voxels with an average above-chance reconstruction performance for both the stimulus and the report (thresholded at *p* < 0.001, uncorrected).

##### 2.7.6.1. Interaction between coherence level and reconstructed labels

We wanted to clarify whether the coherence level affected the stimulus and the report reconstruction in the voxels that code for both. For this, we computed the mean reconstruction performance of the ROI for each coherence level and each reconstructed label. Finally, we performed a repeated-measures ANOVA to test for an interaction effect between coherence level and reconstructed label.

##### 2.7.6.2. Model generalization

To further explore the similarity between the encoding of stimulus and report information in the brain, we performed a series of cross-prediction analyses. The principle of this analyses is the same as in other examples of cross-classification (Cichy et al., 2012; Kaplan et al., 2015; Levine and Schwarzbach, 2017). It involves the use of the GPR estimated in one condition (e.g., the report model estimated with the data acquired in the 0% coherence condition) to predict the data of a different one (e.g., the stimulus directions at 100% coherence condition). If the model generalizes, then there is evidence that the pattern of brain activity is similar across the two conditions. For each subject we tested how well the report model estimated in the 0% coherence condition (which is independent of the physical stimulus) would allow to predict the stimulus identity in each coherence level and vice versa (averaged). The procedure was similar to the one described in “2.7.5.2. Voxel-wise GPR estimation” and Searchlight based stimulus and report reconstruction. However, we ran the analysis on a limited number of voxels (see above). Since we were primarily interested in the generalization of the report model estimated at 0% coherence to the stimulus model estimated at 100% coherence (see “4. Discussion”), we tested whether the generalization performance of this pair of conditions were above chance by computing a one tailed *t*-test.

## 3. Results

### 3.1. Behavioral results

**FIGURE 2:**
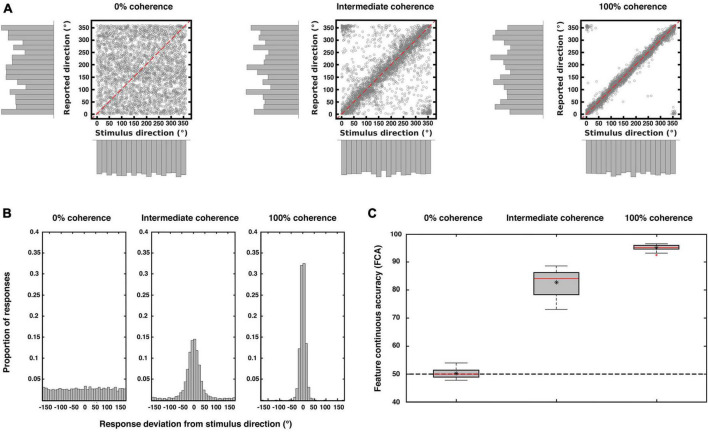
Behavioral results. The three plots show the behavioral performance pooled from all subjects (*N* = 23) for each coherence level. **(A)** Scatterplots and marginal distributions of the stimulus and reported directions for each level of coherence (each point corresponds to one trial). **(B)** Distribution of participants’ response deviations (where 0° corresponds to perfect performance). Note the flat distribution when direction information is absent (0% coherence) and the narrowing of the response distributions with increasing coherence. **(C)** Performance accuracy (measured as deviations as in **(B)** but replotted as FCA, which is scaled such that 100 is perfect performance and 50 is chance performance; see “2. Materials and methods”). In each box, the central red line indicates the median accuracy, the asterisk indicates the mean accuracy. The bottom and top edges of the box indicate the 25 and 75th percentiles, respectively. The whiskers extend to the most extreme data points not considered outliers, and the outliers are plotted individually using a red cross. The dashed black line indicates chance performance. There was a main effect of coherence on accuracy [repeated measures ANOVA; *F*(1.26, 27.818) = 1,357.545; *p* < 0.001; Greenhouse-Geisser corrected for non-sphericity].

Participants were able to correctly perform the continuous task with increasing accuracy the higher the coherence of the stimuli (Figure 2). When sensory information was absent (0% coherence) there was no relationship between direction and report, thus as expected participants responded largely randomly (Figures 2A, B left panels). With increasing sensory evidence the distributions of responses became narrower and centered around the veridical direction (Figures 2A, B mid and right panels). This was also reflected in increasing levels of accuracy with increasing coherence (Figure 2C). As expected from previous work (Töpfer et al., 2022) our chosen report method (see “2. Materials and methods”) minimized the reporting biases across the motion directions.

### 3.2. fMRI results

**FIGURE 3:**
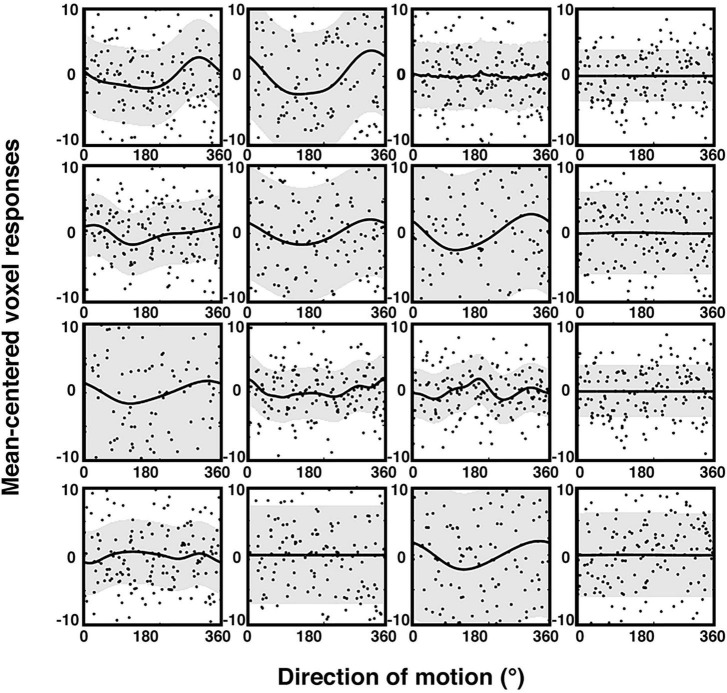
Trial-wise activity and estimated sensory voxel tuning response profiles based on GPR (see “2. Materials and Methods”) for encoding of physical stimulus direction. The plot shows the trial-wise fMRI response (black dots) as a function of motion direction, along with the estimated GPR-based tuning functions of 16 voxels (mean = black lines; standard deviation = gray bands; example participant; 100% coherence condition). The voxels were randomly chosen from the subject’s most informative searchlight of the ROI described in the exploratory analyses (see “2.7.6. Additional exploratory analyses” in the “2. Materials and methods” section). Trial-wise activity (*y*-axis) is here plotted as parameter estimates of a trial-wise 1st level GLM versus corresponding stimulus motion directions (*x*-axis). The estimated response profiles (thick lines, gray regions) were obtained by averaging the voxel-wise GPR predictions across 10 cross-validation folds. The data were mean-centered and are shown at the same scale to facilitate comparison.

The brain signals associated with behavioral judgements were entered into two different preregistered encoding model analyses: one that assessed encoding of the physical stimulus direction, and the other that modeled the encoding of the participants’ trial-by-trial reports. Our model extends the framework of so-called choice probabilities that were primarily developed for binary choices (Britten et al., 1996; Chicharro et al., 2021) to continuous encoding. In comparison to other studies, our encoding models were based on a cyclic version of Gaussian process regression (GPR; Rasmussen and Williams, 2005; Caywood et al., 2017; Dimitrova et al., 2020). This has the advantage of providing not only an estimate of the mean response in each voxel for a given direction, but also of the distribution across trials, separately for each direction, allowing to obtain tuning response profiles (see “2. Materials and methods” for details). Also, this approach does not require any *a priori* assumptions regarding the smoothness of the tuning functions (compared to e.g., Brouwer and Heeger, 2009). Figure 3 shows examples of these voxel tuning functions for sixteen visually responsive voxels of one participant. There is considerable variability with some voxels showing various forms of smoothly varying tuning functions, while others are non-informative and flat. Note that these tuning functions cannot be directly interpreted in terms of single-neuron tuning, but reflect a complex integration of a population of tuned neurons within a voxel (Kriegeskorte et al., 2010; Ramírez et al., 2014; Sprague et al., 2018, 2019; Gardner and Liu, 2019).

#### 3.2.1. ROI results

**FIGURE 4:**
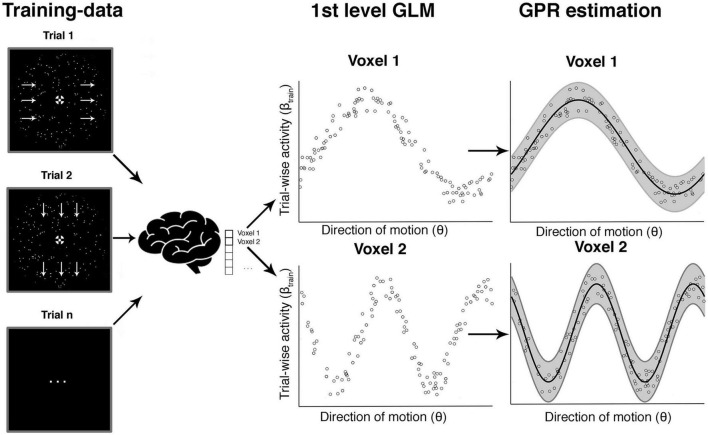
Voxel-wise GPR estimation to obtain tuning response profiles. This schematic example is based on simulated data and illustrates the steps involved in the voxel-wise GPR estimation. For a given direction of motion in a given trial we obtain a (trial-wise) parameter estimate for the response amplitude in each voxel. The middle shows a set of trial-wise measurements for two example voxels. The *x*-axis corresponds to the direction of motion in a trial and the *y*-axis to the trial-wise activity in that voxel. Each vertical section is equivalent to a likelihood function of a brain response amplitude in a voxel given a certain stimulus direction. We use Gaussian process regression (GPR) to estimate the family of these likelihood functions across different movement directions (right column, “GPR estimation”). The analysis can be performed either with the veridical stimulus motion direction **θ_s_** or the reported direction **θ_r_**.

**FIGURE 5:**
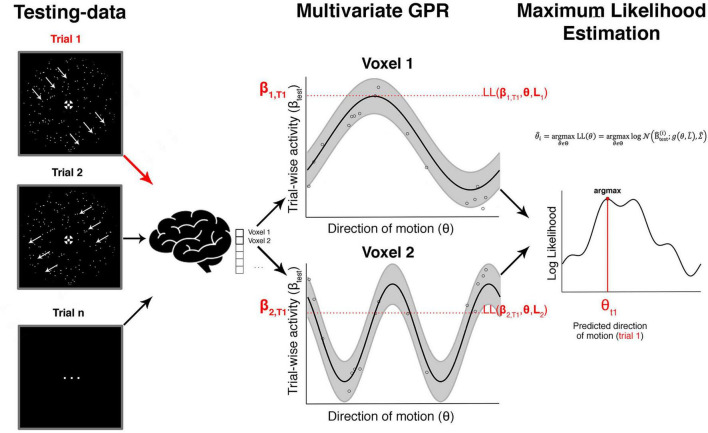
Searchlight-based GPR reconstruction from a multivoxel ensemble of tuning response profiles. This shows the steps involved in the searchlight-based reconstruction of one trial focusing on just two voxels. The red arrow on the left shows an unknown true direction on a single trial T_1_. The aim is to estimate this true direction with maximum precision from an ensemble of trial-wise brain responses using the tuning response profiles. The red parameters on the *y*-axis in the middle column (β_1, T1_ and β_2, T1_) show the measured brain responses in that trial in the two voxels. The horizontal red section illustrates the likelihood values for each direction. If one were only using a single voxel one would estimate the direction as that where the likelihood is maximal (assuming a flat prior as in our study). In our case we combine the likelihood estimates across an ensemble of voxels (in a searchlight) and identify the maximal value, which corresponds to our decoded direction of motion in that trial.

In the next step, we used ensembles of voxel-wise tuning functions from different pre-defined ROIs to reconstruct (a) the veridical motion direction and (b) the reported direction in each given trial (see Figures 4, 5 for details on methods). These five bilateral ROIs were early visual cortex (EVC), MT+, superior parietal cortex (SPC), intraparietal sulcus (IPS), and inferior parietal cortex (IPC). These areas have been reported in previous work to be involved in encoding of motion and decision signals (Kamitani and Tong, 2006; Serences and Boynton, 2007; Hebart et al., 2012; Bode et al., 2013; Levine and Schwarzbach, 2017).

**FIGURE 6:**
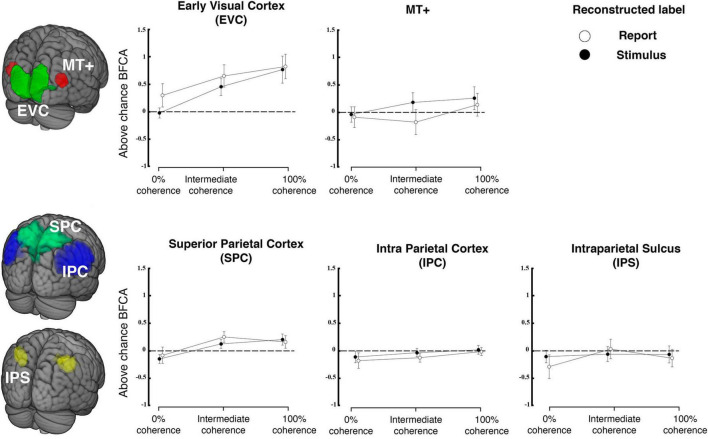
Reconstruction from regions of interest in visual and parietal cortex. The top row shows reconstruction accuracy (expressed as balanced feature continuous accuracy, BFCA, which is corrected for biases due to unequal distribution of responses across the sensory continuum, see “2. Materials and methods”) for regions of interest in early visual cortex (comprising V1, V2 and V3) and MT+ separately for each coherence level. The bottom row shows the same for three regions of parietal cortex. Filled versus open symbols indicate reconstruction of stimulus and report, respectively. Error bars are standard errors. Dashed lines indicate chance level (*N* = 23).

Figure 6 shows the stimulus-related and report-related accuracies for each of these ROIs and each coherence level. For each ROI, we tested whether coherence or label (stimulus versus report) influenced accuracy. Based on previous research on motion direction decoding with fMRI we expected that fMRI-signals would carry highest information in early visual cortex (Kamitani and Tong, 2006; Serences and Boynton, 2007; Hebart et al., 2012), whereas it was unclear whether this region is expected to encode choice (Ress and Heeger, 2003; Brouwer and van Ee, 2007; Serences and Boynton, 2007; Krishna et al., 2021). As expected, we found a main effect of coherence on reconstruction performance for the visual ROI [coherence on EVC: *F*(2, 44) = 11.909, *p* < 0.001] but there was no difference between accuracies for stimuli and reports and no interaction [*F*(1,22) = 2.42, *p* = 0.134; label * coherence *F*(2,44) = 0.69, *p* = 0.507]. A post-hoc *t*-test performed on EVC revealed no difference between the stimulus and the report reconstruction at 0% coherence (*t* = −1.793; *p* = 1, Bonferroni corrected for a family of 15 multiple comparisons).

As in some (but not all) previous studies we also expected the motion sensitive complex MT+ to be informative of veridical motion direction (Kamitani and Tong, 2006) and of perceptual judgements (Serences and Boynton, 2007). We also expected a main effect of coherence on stimulus and report reconstruction (Britten et al., 1996). In contrast to this prediction, the results of a repeated measures ANOVA revealed no effect of coherence in MT+ [*F*(2, 44) = 0.664, *p* = 0.52], no effect of which label was being reconstructed [label on MT+: *F*(1,22) = 2.72, *p* = 0.113] and no interaction [label * coherence on MT+: *F*(2,44) = 0.628, *p* = 0.538]. Additional post-hoc *t*-tests revealed that the mean stimulus reconstruction performance at 100% coherence was not different from that of 0% coherence, in which the stimulus has no net direction of motion (*t* = 1.069; *p* = 1, Bonferroni corrected for a family of 15 multiple comparisons). Please note that these differences to previous work might reflect the fact that we employed continuous stimuli and also that we used a very different response format that did not involve differential or even dispositional motor preparation.

We also found a main effect of coherence on reconstruction performance for one region of parietal cortex [SPC; coherence SPC: *F*(2,44) = 4.219, *p* = 0.021], but no effect of which label was reconstructed [label SPC: *F*(1,22) = 0.712, *p* = 0.408] and no interactions were found [label * coherence SPC: *F*(2,44) = 0.502, *p* = 0.609]. We did not find any effect on reconstruction performance for the other parietal ROIs (IPS and IPC).

### 3.3. Additional exploratory analyses

We then conducted an exploratory analysis aimed to identify informative regions beyond our pre-defined regional hypotheses. For this, we used whole-brain searchlight reconstruction maps (see “2. Materials and methods”). In line with our ROI-based findings this only revealed a cluster in early visual cortex (left occipital pole; see Supplementary material). Interestingly, we found no region where coherence affected reconstruction of choices (see Supplementary material).

Taken together, the early visual cortex is the only area where we were able to reconstruct information about continuous motion stimuli and their corresponding choices.

#### 3.3.1. Interaction between coherence level and reconstructed labels

**FIGURE 7:**
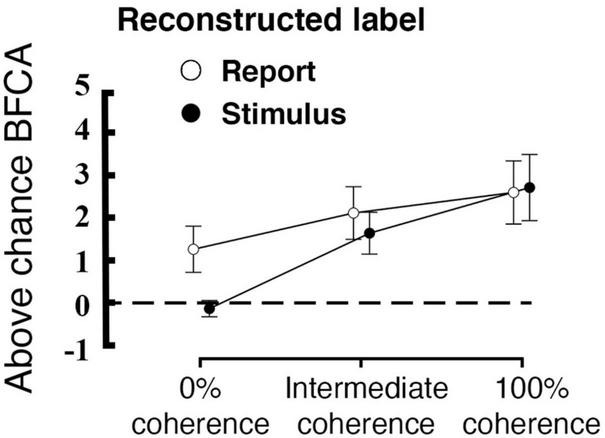
Interaction between coherence level and reconstructed label within a subsection of the early visual region (the conjunction of voxels that code for both stimulus and report). The plot displays the averaged above-chance accuracy (expressed as BFCA, see “2. Materials and methods”) across voxels for each coherence level and each reconstructed label, error bars are standard errors, the black dashed line represent chance level (*N* = 23). Please note that this voxel selection does not affect the difference between encoding of stimulus versus reports.

At 0% coherence we found no evidence for either stimulus encoding (as expected) or response encoding in our early visual ROI. In contrast, other studies have revealed choice signals already in early visual areas (Ress and Heeger, 2003; Serences and Boynton, 2007; Sousa et al., 2021). We thus assessed whether our *a priori* defined early visual ROI might be defined too widely to reveal potential differences between stimulus and report reconstruction. For this, we conducted a single test with a more focused analysis of the voxels that encoded both stimulus and report across all of the coherence levels (0%, Intermediate and 100%). Such voxels were located in the occipital cortex bilaterally. We found a label * coherence interaction effect on the reconstruction performance in these areas [*F*(1.327, 29.191) = 4.426, *p* = 0.034; Greenhouse-Geisser correction for non-sphericity; see Figure 7]. Moreover, post-hoc *t*-tests revealed a significant difference between stimulus and report reconstruction at 0% coherence for this more focused voxel set (Table 1; *t* = 3.560; *p* = 0.011, Bonferroni corrected for a family of 15 multiple comparisons). Please note that the voxel selection for this analysis was collapsed across both stimulus and report reconstruction and was thus not biased *a priori* to yield such a difference.

**TABLE 1:** *Post-hoc t*-tests performed on the stimulus and report reconstruction performance.

		Mean difference	SE	*t*	P_bonf_
Post hoc comparisons—coherence * reconstructed label					
Coherence 0%, report	Coherence intermediate, report	−0.854	0.508	−1.682	1.000
	Coherence 100%, report	−1.338	0.508	−2.635	0.155
	Coherence 0%, stimulus	1.397	0.392	3.560	0.011*
	Coherence intermediate, stimulus	−0.379	0.530	−0.715	1.000
	Coherence 100%, stimulus	−1.453	0.530	−2.744	0.114
Coherence intermediate, report	Coherence 100%, report	−0.484	0.508	−0.953	1.000
	Coherence 0%, stimulus	2.251	0.530	4.250	< 0.001***
	Coherence intermediate, stimulus	0.475	0.392	1.211	1.000
	Coherence 100%, stimulus	−0.599	0.530	−1.131	1.000
Coherence 100%, report	Coherence 0%, stimulus	2.735	0.530	5.164	< 0.001***
	Coherence intermediate, stimulus	0.959	0.530	1.810	1.000
	Coherence 100%, stimulus	−0.115	0.392	−0.294	1.000
Coherence 0%, stimulus	Coherence intermediate, stimulus	−1.776	0.508	−3.498	0.012*
	Coherence 100%, stimulus	−2.850	0.508	−5.613	< 0.001***
Coherence intermediate, stimulus	Coherence 100%, stimulus	−1.074	0.508	−2.116	0.568

*P*-value adjusted for comparing a family of 15.

*p < 0.05, ***p < 0.001.

Please, note that in the 0% coherence condition, the stimulus labels are unrelated with the motion direction (motion signal is absent, and labels are randomly assigned), hence the chance-level reconstruction performance is to be expected.

#### 3.3.2. Model generalization

**FIGURE 8:**
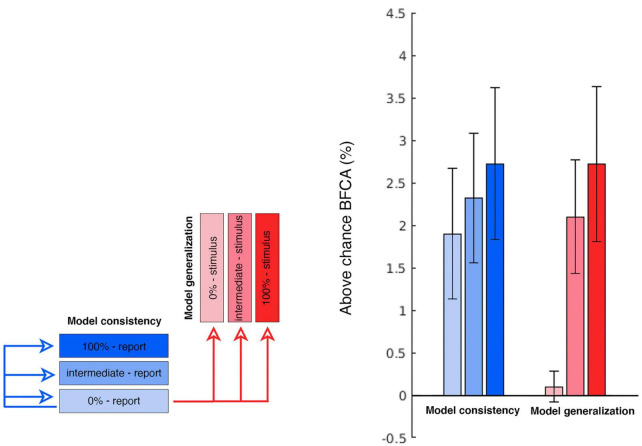
Cross-prediction performances. The figure summarizes the cross-prediction analyses performed to test the model generalization and model consistency. Each bar displays the above-chance generalization accuracy (expressed as BFCA, see “2. Materials and methods”) of the 0% report report-related GPR model to every other condition. The bars on the right hand of the plot (in red) indicate how well the 0% report model allows to reconstruct the stimulus identity in each coherence condition (model generalization). We were primarily interested in observing the generalization performance of the 0% coherence report model to the 100% stimulus (first bar on the right). Also note that since the 0% coherence stimulus model is based on random directions (unrelated with the report or the stimulus), it represents here a form of control condition in which chance-level generalization performance is to be expected (the first red bar in the right group). The group of bars on the left hand of the plot (in blue) illustrates how well the 0% report model allow to reconstruct behavioral reports in each coherence condition (model consistency) (*N* = 23; errorbars: SEM).

In order to further test these choice-related signals, we performed a specifically targeted cross-prediction analysis. We took the report-related reconstruction model estimated at the 0% coherence condition and used it to cross-predict the stimuli in the 100% coherence condition. This model generalization constitutes an independent test of the choice signals. Importantly, by training the reconstruction model on the 0% condition we avoid that our model is influenced by residual sensory information. This tests whether the choices at 0% coherence, i.e., when participants are guessing, use a similar representational format as the encoding of stimuli. Indeed, the 0% coherence report model generalized to the 100% stimulus condition (and vice-versa) [right tailed one sample *t*-test; *t*(22) = 2.969; *p* = 0.004]. For exploratory purposes we also repeated this procedure with stimulus motion directions in the intermediate and 0% coherence condition. The generalization tests between the different reporting conditions constitute a test of model consistency across evidence levels and were all above chance. As predicted the cross-prediction was significant between 0% coherence report and the two above-chance stimulus encoding conditions, but not the 0% coherence condition where there is no sensory information. The summary of this cross-prediction analysis is displayed in Figure 8. Thus, we conclude that choice-related signals are present at guessing levels in early visual cortex and that these signals are encoded in a similar form as the physical stimulus features.

#### 3.3.3. Eye movements

In order to control for involuntary eye movements that might have confounded our reconstruction analyses, we had first decided to exclude trials where subjects exceed a threshold of 2 dva for more than 200 ms during the stimulation period. However, recent evidence indicates that involuntary eye movements below our adopted threshold might still affect brain activity, thus constituting a potential confound (Merriam et al., 2013; Thielen et al., 2019). For this reason, we complemented our preregistered results with an additional analysis that exploited the same GPR-based estimation and reconstruction techniques adopted for the analysis of brain signals, to the eye movements recorded with eye-tracker (see Supplementary material). Our results revealed that the pattern of eye movement was unrelated to the stimulus or the report in the 100% and the intermediate coherence condition, but it was predictive of participants’ reports in the 0% coherence condition (see Supplementary material). Thus, it could have been possible that the choice signals in the guessing condition were partly affected by eye movements. However, this would not explain why our report model generalized from the guessing to the other conditions, where eye movements did not play a role.

## 4. Discussion

Previous studies on neural mechanisms of perceptual decision making have often focused on simple decisions involving discrete alternatives. These have found discrete decisions to be encoded either in intention-related formats (Shadlen et al., 2008), or in high-level but effector-independent formats (Heekeren et al., 2008; Bennur and Gold, 2011; Hebart et al., 2012; Park et al., 2014; Brincat et al., 2018). However, it has remained unclear how the brain encodes perceptual choices regarding an entire continuum of features, which is what our study aimed to address (see also Nichols and Newsome, 2002; van Bergen et al., 2015; Ratcliff, 2018 for other examples of continuous stimuli). Our study also disentangled stimulus and choice-related activity from motor responses by using a visual comparison stimulus (a rotating bar). Our modeling of single-voxel responses as continuously varying distributions using Gaussian process regression (GPR; Rasmussen and Williams, 2005) allowed us to estimate tuning functions in considerable detail and without making *a priori* assumptions regarding their smoothness.

Our design revealed that the activity of visual voxels was modulated by both the stimulus directions across trials (Figure 3) and by participants’ perceptual choices regarding the motion direction. We were able to reconstruct the stimulus motion direction from clusters of voxels in early visual cortex (Figure 6), and to identify a region of occipital cortex in which the change in coherence level had a different effect on stimulus and report reconstruction (Figure 7). These findings extend previous research on decoding of visual motion direction and perceptual choices from brain signals (Kamitani and Tong, 2006; Serences and Boynton, 2007; Hebart et al., 2012) to the feature-continuous case. Moreover, they indicate that perceptual decisions can be represented by neural populations encoding the stimulus distribution over a continuous feature space in early visual areas (Beck et al., 2008; Smith, 2016; Ratcliff, 2018). By effectively using such distributions for quantifying the degree to which trial-by-trial variations in brain signals are predictive of the corresponding judgements, we adapted the framework of so-called choice probabilities (Britten et al., 1996; Chicharro et al., 2021) to a feature-continuous task.

Our encoding models also allowed us to go beyond studying encoding itself and to explicitly assess the similarity between the stimulus-encoding and the report-encoding. For this we tested whether the information encoded by the report model when guessing could be used to infer the stimulus motion direction in a set of cross-prediction analyses (Figure 8). This procedure relies on the assumption that if the model estimated during one condition allows to predict data from another, then the brain activity patterns elicited by the two conditions are similar (Cichy et al., 2012; Kaplan et al., 2015; Levine and Schwarzbach, 2017). Note that for 100% and intermediate coherence levels there is a strong correspondence between the stimulus and corresponding reports (see e.g., Figure 2B). This would make any correlation between stimulus and response encoding trivial. Thus, we limited our generalization analysis to the “guessing” condition, i.e., the reports for 0% coherence, which were not contaminated by stimulus-related signals. By using information encoded from this report model, we were able to predict the stimulus direction at intermediate and full coherence. We refer to this effect as model generalization. In contrast, we refer to model consistency as the ability of the report to predict reports in other coherence levels, but this is not a key focus here (see Figure 8). Our generalization analysis was successful, thus indicating that the sensory information and the choices are encoded in a similar representational feature space. This would suggest that the neural mechanisms recruited by visual areas to support perceptual decisions are, to some extent, the same used to encode visual stimuli. Whether this generalizes to discrete choice options or to other forms of report remains an open question.

Using our stimuli and task we did not find any significant motion direction or choice-related signal in area MT+. Previous studies have yielded somewhat inconsistent results on what to expect. There are numerous studies indicating the key role of MT+ in motion perception (Newsome and Paré, 1988; Britten et al., 1996; Rees et al., 2000; Braddick et al., 2001). However, our null result is compatible with at least some previous fMRI multivariate pattern analysis studies that have observed comparatively low levels of motion direction information in MT+ (Kamitani and Tong, 2006; Serences and Boynton, 2007; Beckett et al., 2012; Hebart et al., 2012). There might be multiple explanations for this. First, it is possible that the small amount of voxels from MT+ might negatively impact our ability to extract motion related information (Kamitani and Tong, 2006; Serences and Boynton, 2007), even though similar results were obtained by a study employing a 7T scanner, and thus increasing the amount of voxels available (Beckett et al., 2012). We should also consider that the relationship between single-cell tuning and single-voxel BOLD response profiles requires additional assumptions on how the voxel samples populations of tuned sensory neurons (Nevado et al., 2004; Haynes, 2015; Sprague et al., 2018, 2019; Gardner and Liu, 2019). For example, an additional factor might be related to the spatial distribution of directionally selective neurons within MT+, which might lead to small differences in the BOLD signal across directions in that area (Kamitani and Tong, 2006; Beckett et al., 2012; Hebart et al., 2012). It has also been suggested that the motion stimuli employed in these experiments might elicit coarse-scale directional preferences, which might contribute to motion direction decoding from early visual areas (Wang et al., 2014). This might explain why fMRI studies employing classification techniques have found motion direction information to be distributed across multiple visual regions, and the relatively low decoding performance from MT+. Alternatively, it is also possible that the contribution of early visual areas to the encoding of visual motion direction is greater in humans than results from invasive neuronal recordings from non-human primates might suggest, but this remains speculative. Finally, we cannot rule out the possibility that our method is unable to identify tuning information from MT+ due to lack of power. While we complemented our empirical results with a simulation analysis (see Supplementary material) showing the reconstruction performance expected from voxels modulated and those not modulated from motion direction, we do not know the exact form and magnitude of voxel tuning in our empirical data.

We were unable to find stimulus or choice-related information in parietal areas. This result is partly consistent with previous fMRI studies involving RDKs that attempted to dissociate motor preparation from perceptual choices (Serences and Boynton, 2007; Liu and Pleskac, 2011; Hebart et al., 2012). Serences and Boynton (2007), for example, did not find any choice-related information in parietal areas even in the ambiguous motion condition, whereas Hebart et al. (2012) were able to decode perceptual choices from the posterior parietal cortex in the 0% coherence condition. It is important to note, however, that these studies have adopted binary choices and participants expressed their decisions by using mutually exclusive categories. While we cannot exclude that the parietal cortex might be involved in a form of categorical decision-making (Freedman and Assad, 2011), we found no evidence of continuous choice representation in these areas.

There are two other factors besides the continuous decisions that might help explain why choice-related information is only observed in early visual areas with our design. First, in our task specific motor preparation is impossible due to the employment of a visual comparison stimulus that rotated independently. While some studies have decorrelated choices and specific motor commands (e.g., Bennur and Gold, 2011; Bode et al., 2013) this is typically done by post-cueing a variable stimulus-response mapping and thus, the preparation of the response can be at least conditionally prepared (i.e., the motor command can be prepared in form of a differential response to a mapping cue).

A second difference is that the nature of our task involves a brief delay between the perceptual decision and the report in the absence of any possibility for motor preparation. Solving such a task might be achieved by briefly memorizing the target stimulus. In human neuroimaging studies of visual working memory sensory regions have been shown to encode stimulus features across delays (Serences et al., 2009; Riggall and Postle, 2012; Pratte and Tong, 2014; Christophel et al., 2017; but see also Harrison and Bays, 2018), which is consistent with neuroimaging evidence from feature-continuous perceptual tasks involving a working memory component (van Bergen et al., 2015). This might raise the concern that our results reflect maintenance-related brain activity rather than perceptual decision-making. We cannot completely rule out that participants make their decision before the end of the stimulus presentation and keep it in mind until the motor response: in this case, at least part of the modeled brain activity could be related to decision maintenance. Here, we attempted to minimize the potential influence of maintenance-related signals by focusing on the analysis of choice-related neural activity happening during the stimulus presentation, rather than on the subsequent delay period. This is substantially different from working memory experiments using fMRI to study the effects of maintenance on brain activity, where the analyses focus on delay periods which typically last longer than our stimulus presentation time (e.g., Li et al., 2022, for a meta-analysis).

To summarize, our combination of a continuous feature task and fMRI encoding models suggested that early visual areas, but not MT+, allowed to reconstruct both continuous physical motion stimuli as well as continuous choices. Taken together, our results indicate that perceptual decisions regarding continuous sensory features might be encoded in early visual areas, potentially akin to visual working memory signals in sensory areas.

## Data availability statement

The datasets presented in this study can be found in online repositories. The names of the repository/repositories and accession number(s) can be found below: OSF: https://osf.io/vcmdg/; Github: https://github.com/RiccardoBarb/GPR_fMRI.

## Ethics statement

The studies involving humans were approved by the Ethics Committee of the Humboldt University of Berlin. The studies were conducted in accordance with the local legislation and institutional requirements. The participants provided their written informed consent to participate in this study.

## Author contributions

RB: Conceptualization, Data curation, Formal analysis, Investigation, Methodology, Software, Validation, Visualization, Writing – original draft, Writing – review and editing. FT: Conceptualization, Data curation, Formal analysis, Investigation, Methodology, Software, Validation, Visualization, Writing – review and editing. JS: Formal analysis, Methodology, Software, Supervision, Validation, Writing – review and editing. CB: Project administration, Supervision, Validation, Writing – review and editing, Methodology. HS: Conceptualization, Methodology, Supervision, Validation, Writing – review and editing. J-DH: Conceptualization, Funding acquisition, Methodology, Project administration, Resources, Supervision, Validation, Writing – review and editing, Writing – original draft.
