# Supplementary material for: Encoding of continuous perceptual choices in human early visual cortex
Source: Front Hum Neurosci. 2023 Nov 13;17:1277539. doi: 10.3389/fnhum.2023.1277539 (PMC10679739; doi:10.3389/fnhum.2023.1277539)
Supplement: Supplementary file 1 [file Data_Sheet_1.docx]

Supplementary Material

# Additional exploratory analyses

## searchlight-based reconstructions and effect of coherence

We also performed additional whole-brain searchlight decoding to identify regions potentially outside our pre-defined ROIs that might encode stimulus-related or choice-related information. For this, the single-subject BFCA maps resulting from the searchlight reconstructions were smoothed using a Gaussian kernel with a FWHM of 6 mm and spatially normalized with SPM12. In order to identify searchlights in which the reconstruction performance was significantly above chance (i.e. BFCA > 50%), we used two one-way factorial designs (one for stimulus and one for report) with coherence level as a within-subject factor. For each model, we specified three t-contrasts. In this way we could identify clusters with significant above-chance information for each coherence level. We expected the reconstruction performance of searchlights located in visual areas, to decrease as a function of decreasing coherence. We also expected to identify clusters of voxels carrying information about perceptual judgements in visual (Britten et al., 1996; Serences & Boynton, 2007; Hebart et al., 2012; Sousa et al., 2021) and parietal areas (Gold & Shadlen, 2007; Brincat et al., 2018; Hebart et al., 2012, 2016; Levine & Schwarzbach, 2017). We further predicted the coherence level to have an effect on report reconstruction performance as well. We evaluated the effect of coherence on the reconstruction performance by inclusively masking the voxels that showed an average effect of reconstruction across coherence levels (p < 0.001, uncorrected). This procedure was done separately for stimulus reconstruction and report reconstruction. Please note that this analysis is not circular because the test for an effect of coherence is orthogonal to the test for average reconstruction performance.

The whole-brain searchlight reconstruction at 100% coherence revealed stimulus information from voxel clusters located in the left (FWE_c_, p < 0.05, K = 1677; cluster-defining threshold p < 0.001) and in the right occipital cortex (FWE_c_, p < 0.05, K = 1024; cluster-defining threshold p<0.001). For the intermediate coherence condition and for the 0% coherence condition, we found no searchlights that were significantly predictive of the stimulus motion direction. Note that in the 0% coherence condition, the stimulus has no global motion direction, thus a chance-level reconstruction performance is to be expected. In the left and right occipital cortex, we also found clusters of voxels informative about participants’ reports for the 100% coherence condition (left: FWE_c_, p < 0.05, K = 1049; cluster-defining threshold p < 0.001; right: FWE_c_, p < 0.05, K = 864; cluster-defining threshold p < 0.001) as well as for the intermediate coherence condition (left: FWE_c_, p < 0.05, K = 201; cluster-defining threshold p<0.001; right: FWE_c_, p < 0.05, K = 487; cluster-defining threshold p<0.001). For the 0% coherence condition, we were not able to identify clusters informative about participants’ reports (Supplementary Figure 1).

Since neurons in early and extrastriate visual areas are tuned to motion directions (Albright et al. 1984; Movshon & Newsome, 1996; Nichols & Newsome, 2002), we reasoned that if population-level measurements of neural activity obtained from single voxels reflects this property (Nevado et al., 2004; Haynes, 2015; Sprague et al., 2018), the stimulus reconstruction performance should be maximum in the 100% coherence condition, and progressively decrease at intermediate coherence. At 0% coherence instead, the stimulus has no net motion direction and the reconstruction performance should be at chance level. We therefore expected to identify a main effect of coherence on the stimulus reconstruction performance in visual areas. We identified a cluster of voxels located in the left occipital pole, where coherence level had an effect on the performance in stimulus reconstruction (FWE_c_, p < 0.05, K = 262; cluster-defining threshold p<0.001). Similarly, we predicted that a possible effect of coherence on the report reconstruction might be present, and driven by the expected correlation between the stimulus identity and participants’ report, when the stimulus is clearly visible. However, we did not find clusters that showed such effect for the report reconstruction performance (Supplementary Figure 2).

## Control analysis: stimulus and report reconstruction from eye-tracking data.

The use of motion stimuli such as our RDK might trigger involuntary eye movement (Cohen et al., 1977) that can be informative about the direction of perceived motion (Wilbertz et al., 2018). Eye movements also have an effect on brain activity measured with fMRI and can thus constitute a potential confound (Merriam et al., 2013), even when participants are specifically instructed to maintain fixation (Thielen et al., 2019). For this reason, we tested whether the recorded gaze position (x and y ordinates), was informative about the physical stimulus direction or participants’ perceived direction. For this analysis we used the gaze position of 21 out of 23 subjects who participated in the main fMRI experiment (we couldn’t record the traces of two participants for technical reasons). We reasoned that if the gaze position is systematically correlated with the presented motion direction or with participants’ reports, the x and y ordinates should exhibit a specific position profiles, that can in turn be used to perform stimulus and report reconstruction. In order to check whether the pattern of participants’ eye movements was related with the stimulus or the reported motion directions, we estimated such profiles with a cyclic version of the GPR (see Materials and Methods in the main manuscript) and performed stimulus and report reconstruction at each time point with a procedure similar to the one adopted for the main fMRI analysis (see Materials and Methods).

The preprocessing pipeline employed for this analysis was different from the one performed for fixation control (see Materials and Methods). Blinks, detected by the provided software from Eye Link, were linearly interpolated using the approach described in Urai et al. (2017). The resulting traces were filtered for electronic noise using a Butterworth filter (low cut off 5Hz, high cut off 100Hz) following Thielen et al. (2018). The complete trace of each session was linearly detrended to account for drifts that appear due to the long continuous recordings during each session (each approximately 1.5h). An additional linear detrending was performed separately on each run, to counterbalance slow drifts in head position in the scanner. Periods of interest (500 ms before stimulus onset together with 2000 ms stimulus period) were combined across the two recording sessions. Data from the period of interest were baseline corrected using the pre-stimulus interval (500 ms up to stimulus onset).

After preprocessing, the x and y gaze positions of each subject were grouped by coherence levels (0%, medium, 100% coherence) resulting in a maximum of 160 trials per condition. To reduce computational time, we only considered time points during the stimulus period (2000 ms) and resampled the signal at 50Hz. For each time point we estimated two position-related profiles (one for each ordinate) by entering the trial-wise position value together with the corresponding stimulus motion direction $\theta_{s}$ or the reported direction $\theta_{r}$ into a cyclic version of the GPR. Please note that this procedure is very similar to the one previously described for the estimation of voxel-wise response profiles (see eq. 3 in the Materials and Methods section - where the parameter $\hat{\beta}_{j}$ represents now the trial-wise recorded position of each ordinate in a single time point). The estimation of the position profiles was performed with a leave-one-run-out cross-validation scheme, by only using trials in which participants were maintaining fixation (see Materials and Methods).

The stimulus and report reconstructions were estimated using the same procedure described in the Materials and Methods*.* However, the estimated gaze position profiles instead of voxel response profiles, were used to predict the stimulus direction $\theta_{s}$ or the reported direction $\theta_{r}$ in a run-wise cross-validation procedure. Please note, that in this case it is not necessary to adopt regularization for estimation of the covariance matrix because the number of position profiles (one for x and one for y ordinates) does not exceed the number of trials across runs (see eq. 9 in the Materials and Methods section). The results were evaluated by testing if the averaged BFCA across subjects was above chance for each timepoint. Statistical analyses were corrected for multiple comparison by performing a cluster-based permutation test (Maris & Oostenveld, 2017).

The group-level average reconstruction performance for the stimulus and the report labels are depicted in Supplementary Figure3. We were not able to identify stimulus-related information in any of the three coherence levels. Instead, the evaluation of the report model indicates that the pattern of eye movements was informative about participants’ report in the 0% coherence condition. More precisely we were able to identify clusters of above-chance reconstruction performance, peaking after 1000 ms. Eye movement were not predictive of participant’s choices for the intermediate or 100% coherence levels.

# The problem of feature continuous accuracy with unbalanced labels

In order to evaluate the performance of our GPR-based reconstructions, we implemented a balanced version of FCA (BFCA – see eq. 15 in the *Materials and Methods* section). Our goal was to obtain a measure of performance that could be intuitively compared to a standard accuracy measure with values distributed between 0% and 100%. FCA is derived by rescaling the continuous values of the absolute angular deviation (see eq. 1 and 2 in the Materials and Methods section); see also Pilly & Seitz, 2009), to evaluate the reconstruction performance. The need for a *balanced* version of FCA was due to participants’ responses in the 0% coherence condition being unbalanced (Supplementary Figure 4) as is often the case for reports, even despite the use of our sensory matching approach that minimizes such biases (Töpfer et al., 2022). In case of a standard classification analysis, training and testing a classifier with unbalanced labels make accuracy an unreliable measure of performance (Japkowicz & Stephen, 2002). More specifically, when the performance of a classifier is tested on an imbalanced dataset, it might lead to the misleading finding of significant above-chance performance of the classifier (Brodersen et al., 2010), simply because the classifier tends to reproduce the distribution of the training dataset.

# Comparison between FCA and BFCA

## Simulation analysis

In order to illustrate how BFCA and FCA are related with each other, as well as with the underlying independent variable distribution, we here show a simulation performed on synthetic data. In order to match the features of our experimental design, we simulated a $t \times1$ vector of trial-wise parameter estimates $\hat{\beta}_{j}$ for a total of 1000 voxels where $t=160$ total trials were generated across 10 runs. We also generated a vector $\theta$ corresponding to the independent variable (the stimulus or the report direction).

For the current simulation we distinguished four alternative scenarios:

1. $\boldsymbol{\theta}$ **modulates ${\hat{\boldsymbol{\beta}}}_{\boldsymbol{j}}$ and the distribution of $\boldsymbol{\theta}$ is balanced**. This situation corresponds to the hypothesized behavior of voxels sensitive to motion directions (as the stimulus directions are balanced across runs in our experimental design);
2. $\boldsymbol{\theta}$ **modulates ${\hat{\boldsymbol{\beta}}}_{\boldsymbol{j}}$ and the distribution of $\boldsymbol{\theta}$ is unbalanced**. This scenario corresponds to the hypothesized behavior of voxels sensitive to participants’ reports in our experimental design (as participants’ reports are unbalanced, especially at 0% coherence level);
3. $\boldsymbol{\theta}$ **does not modulate ${\hat{\boldsymbol{\beta}}}_{\boldsymbol{j}}$ and the distribution of $\boldsymbol{\theta}$ is balanced**. This should be the case for voxels insensitive to the stimulus direction. Such voxels should not produce spurious above-chance FCA when combined for searchlight-based reconstruction;
4. $\boldsymbol{\theta}$ **does not modulate ${\hat{\boldsymbol{\beta}}}_{\boldsymbol{j}}$ and the distribution of $\boldsymbol{\theta}$ is unbalanced**. We assume that this scenario could possibly produce spurious above-chance FCA when the voxels are combined for searchlight-based reconstruction.

We applied the same analyses described in the manuscript (see *Materials and Methods*) to estimate voxel-wise response profiles using GPR and to perform the searchlight-based reconstruction using MLE. The simulated searchlights consisted of 241 voxels. We finally evaluated the reconstruction performance by using averaged FCA and BFCA.

## Simulation results

The results of the simulation are summarized in Supplementary Figure 5. We obtained an above-chance reconstruction performance for cases 1 (mean FCA: 92.31%, SD: 1.57; mean BFCA: 91.73%, SD: 1.9) and 2 (mean FCA: 92.72%, SD: 1.09; mean BFCA: 90.99%, SD: 1.91). Interestingly, for case 3 the mean reconstruction performance is around chance for both measures (mean FCA: 50.36% , SD: 2.61; mean BFCA: 49.21%, SD: 2.56) whereas for case 4 the distribution of FCA is skewed toward right (mean FCA: 56.23%, SD: 4.25) whereas BFCA values are not (mean BFCA: 46.98%, SD: 6.06), as confirmed by two one-sample right tailed t-tests evaluating whether the mean values were greater than 50% (FCA > 50% : t = 46.278; p < 0.001; BFCA > 50%: t = -15.747; p = 1).

## Real data analysis

We computed the result the whole-brain searchlight analysis for all of the 23 subjects both with FCA and BFCA as measures of reconstruction performance. The maps were obtained following the procedure described in the *Materials and Methods* section. For the purpose of this analysis we only considered three main conditions:

1. **Stimulus labels at 100% coherence**. In this condition, the stimulus motion directions are balanced, therefore the reconstruction performance should be above chance only for the searchlights with voxels sensitive to motion directions. Because the distribution of the stimulus directions is balanced, we expect no difference between the reconstruction performance computed with FCA and BFCA.
2. **Stimulus labels at 0% coherence**. In this condition, the stimulus had no real motion direction, but each trial was assigned a motion direction, generated according to our randomization scheme (see *Materials and Methods*) This results in a balanced label distribution. Because of this, no searchlight should result in above-chance reconstruction performance. Following the outcome of the simulation described above, we expect no difference between the reconstruction performance computed with FCA and BFCA.
3. **Report labels at 0% coherence**. Here, the labels assigned to each trial correspond to the motion directions reported by participants. Therefore, the distribution of the reports across trials reflects the idiosyncratic biases of each subject. In this condition, because some participants’ choices lead to an unbalanced distribution of reported motion directions, we suspect that FCA leads to spurious above-chance reconstruction performance. Based on the outcome of the simulation, we hypothesize a difference in the reconstruction performance computed with FCA and BFCA.

We then used SPM12 to compare the FCA and the BFCA maps of the 23 participants. We performed three second-level paired t-tests to evaluate our hypotheses.

## Real data results

The results are shown in Supplementary Figure 6. The two measures (FCA and BFCA) were not significantly different when using the stimulus labels at 100% coherence and 0% coherence (Appendix 2 - Figure 3). However, the FCA was significantly different from BFCA when using the labels of the 0% reports in a large cluster covering various portions of the brain (FWE_c_, p < 0.05, K = 667990; cluster-defining voxel threshold p < 0.001). The results remained consistent even when we lowered the cluster-defining threshold to p < 0.0001 or p < 0.00001, with many significant clusters of smaller size scattered throughout the brain reaching significance level (FWE_c_, p < 0.05, smallest K = 70; cluster-defining voxel threshold p < 0.0001; FWE_c_, p < 0.05, smallest K = 20; cluster defining voxel threshold p < 0.00001).

# Supplementary Figures


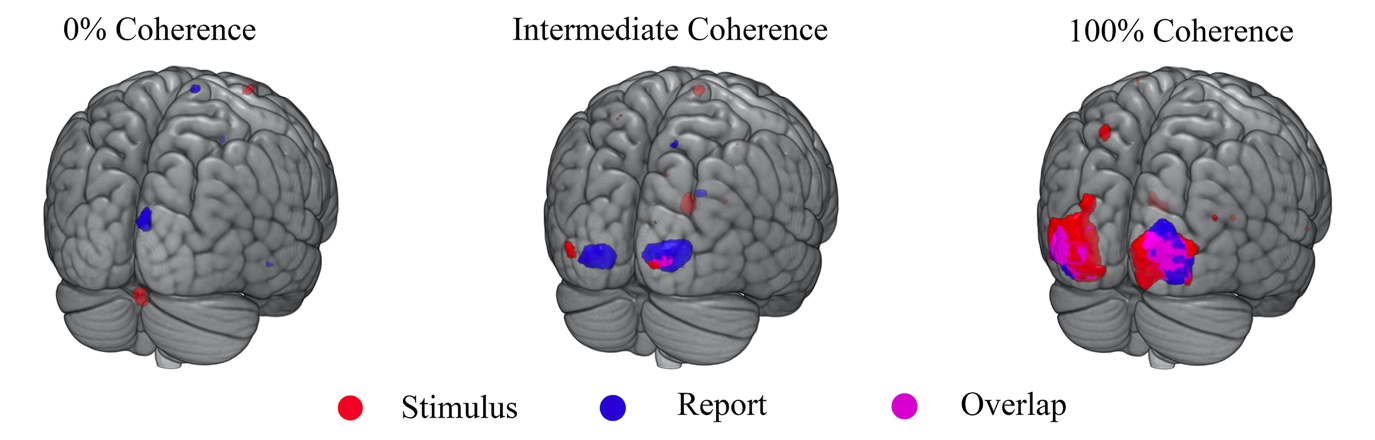


Supplementary Figure 1. Searchlight-based accuracy maps (plotted here for BFCA, see Methods). The images show results of the searchlight-based stimulus and report reconstructions for three coherence levels (left: 0%; middle: intermediate; right: 100%). The searchlights are mapped with different colors: red indicates significantly above-chance reconstruction performance for stimulus, blue indicate above-chance reconstruction performance for report, and purple indicate the overlap between the two. Please note that the maps are shown for display purposes (for visualization thresholded at p < 0.001 and not corrected for multiple comparisons).


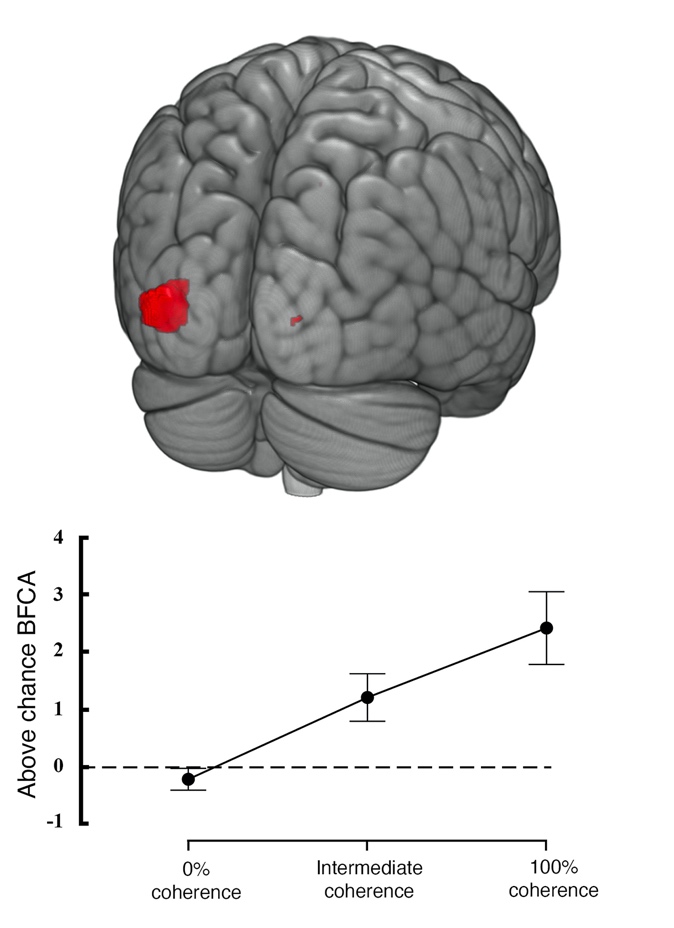


Supplementary Figure 2. Effect of coherence on searchlight-based stimulus reconstruction performance. The picture on top shows clusters of voxels where coherence has a significant effect on stimulus reconstruction performance. The map is thresholded at p < 0.001, uncorrected for multiple comparisons. The plot on the bottom displays the averaged above-chance accuracy (BFCA minus baseline of 50%) extracted from the searchlights in which coherence had a significant effect on stimulus reconstruction performance, error bars are standard errors (N=23).


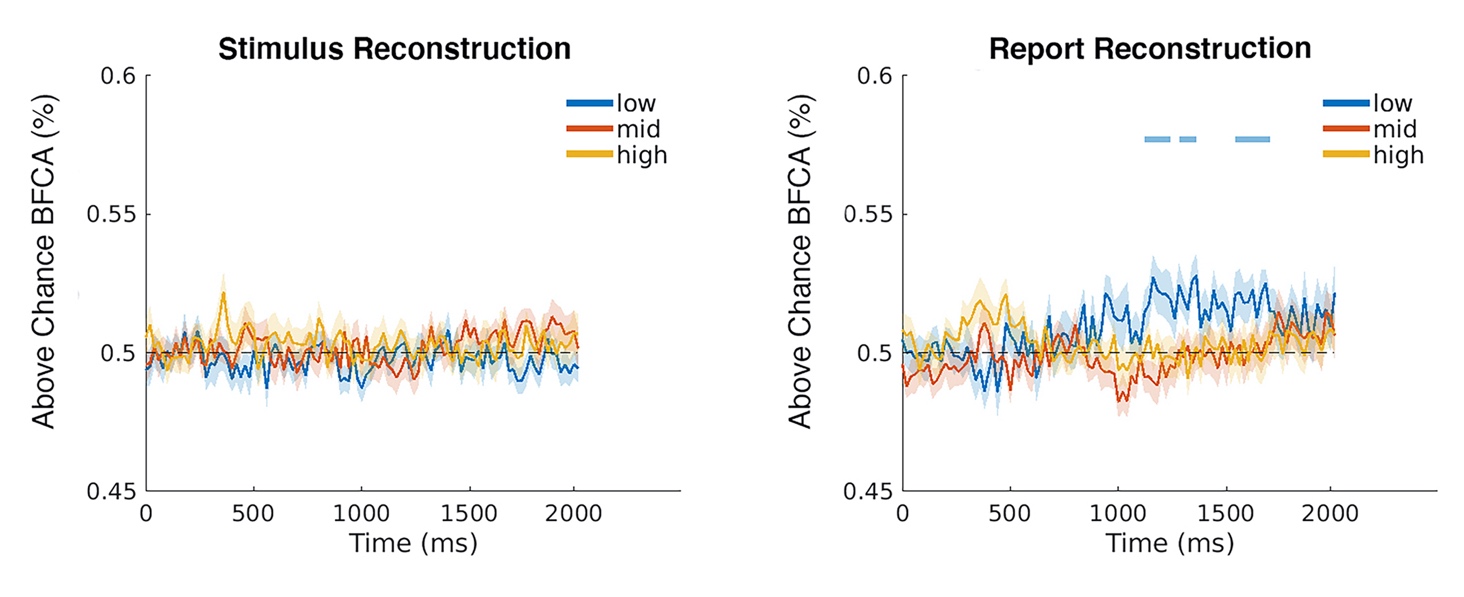


Supplementary Figure 3. Group average (N=21) accuracy (expressed as BFCA, see *Materials and Methods*) at each time point relative to stimulus onset. The upper picture displays the stimulus reconstruction performance, the lower picture shows the report reconstruction performance for three coherence levels (blue: 0%; red: intermediate; orange: 100%). Shading around the individual curves indicates ±1 SEM. The light blue lines on top of the curves depict clusters of time points for which the reconstruction performance was greater than chance after correction for multiple comparisons. Please note that GPR estimated from eye-movements are predictive of the reports for the 0% coherence condition but not of those given at intermediate and 100% coherence. Such result together with those of our model consistency and model generalization analyses (see *Results*), suggest that eye movements were unrelated with the brain signals used to reconstruct participants’ choices in the 0% coherence condition (see *Results*).


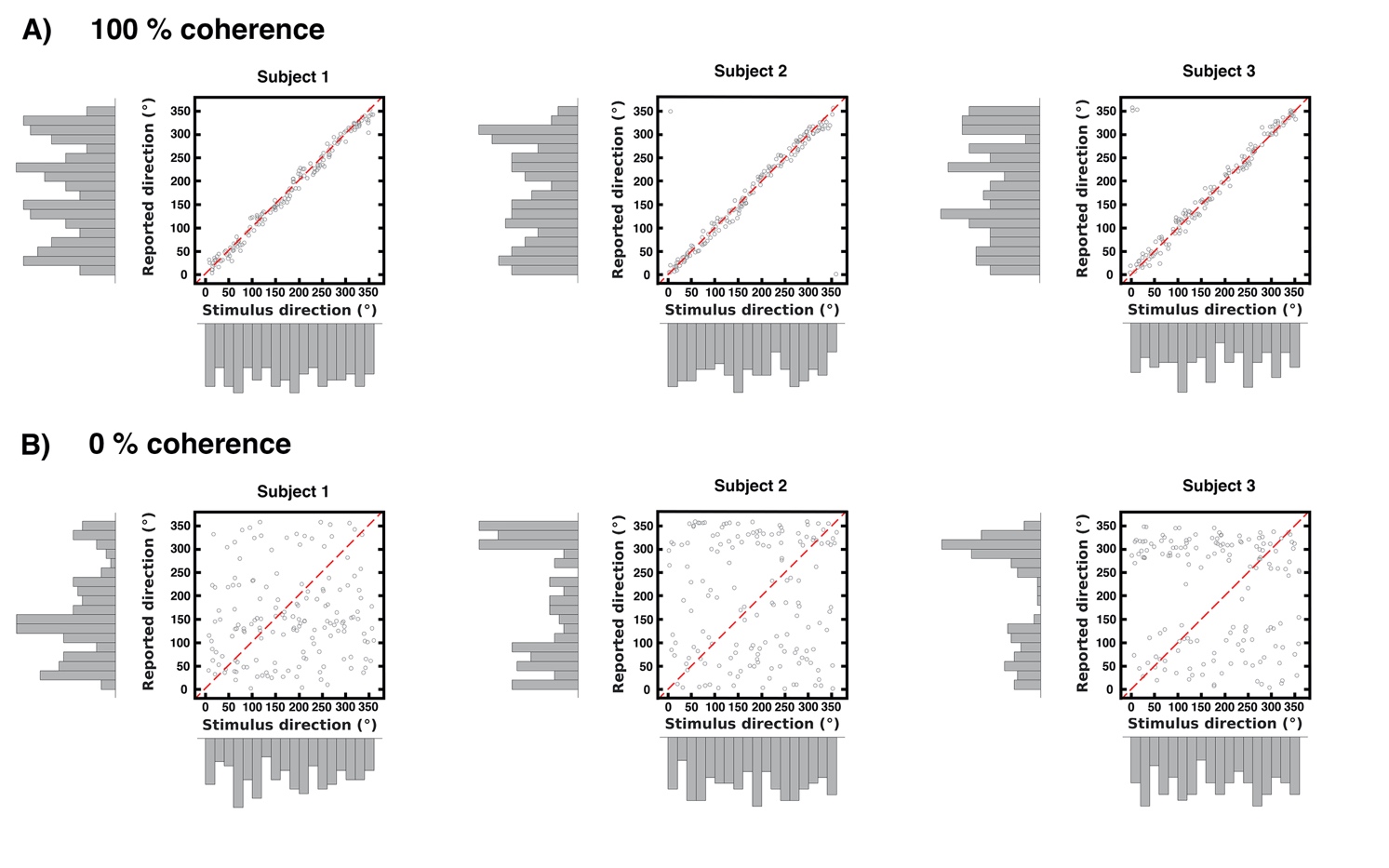


Supplementary Figure 4. The scatterplots display the trial-wise reported direction of 3 example participants against the trial-wise motion direction, with the corresponding marginal distributions. A) Each plot on the top row shows the data distribution obtained from 160 trials in the 100% coherence condition. B) The bottom row shows the data distribution for the 0% coherence condition. Note that in this case the motion direction labels were generated following the randomization scheme described in the manuscript (see the *Materials and Methods* section), as no real motion direction was present in the stimulus.


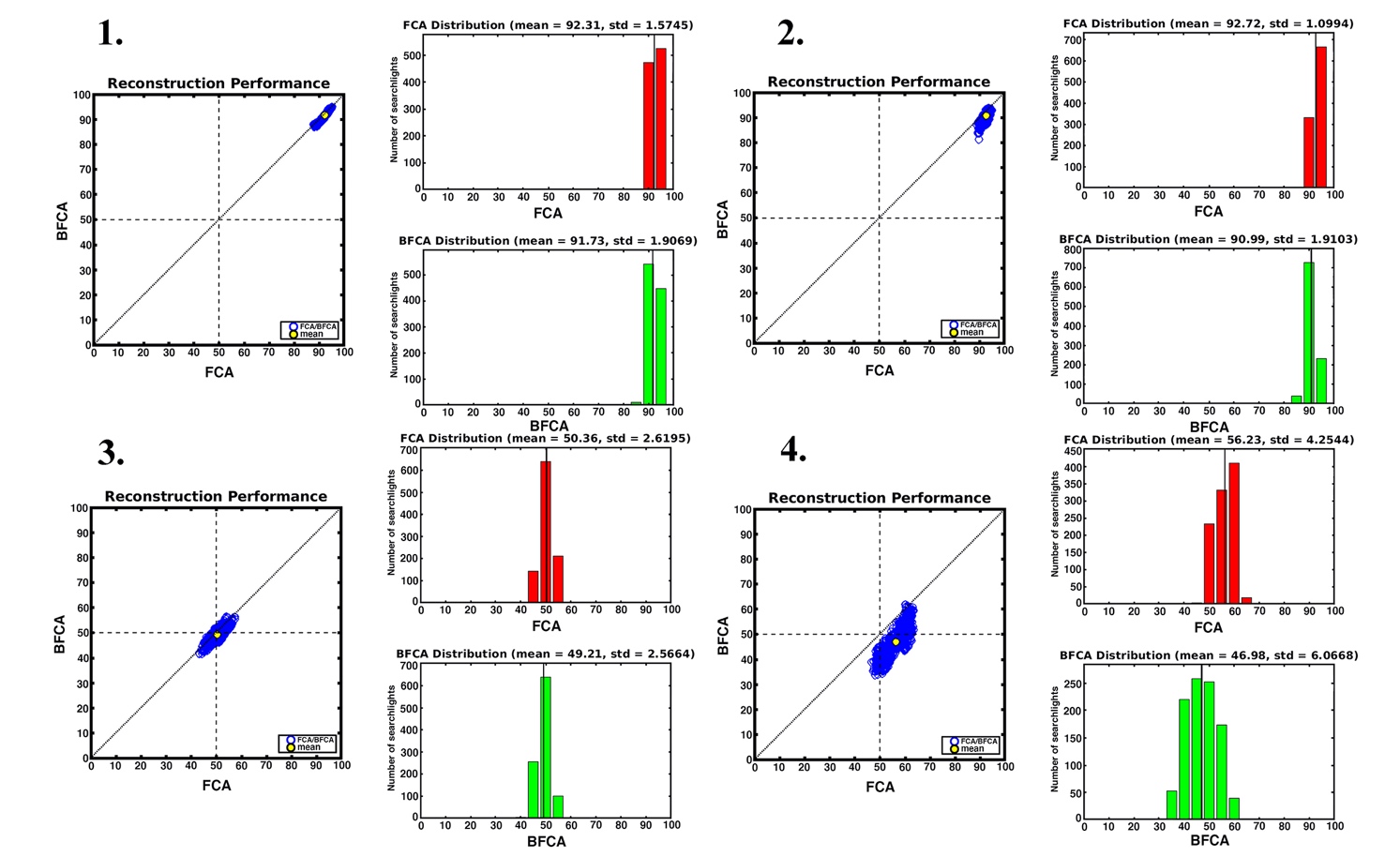


Supplementary Figure 5. Comparison of reconstruction performances obtained with simulated data. The picture illustrates the distribution of FCA (red) and BFCA (green) in the four scenarios examined in the simulation. 1. The relationship between FCA and BFCA for the condition in which $\boldsymbol{\theta}$ modulates ${\hat{\boldsymbol{\beta}}}_{\boldsymbol{j}}$ and the distribution of $\boldsymbol{\theta}$ is balanced. 2. Condition in which $\boldsymbol{\theta}$ modulates ${\hat{\boldsymbol{\beta}}}_{\boldsymbol{j}}$ and the distribution of $\boldsymbol{\theta}$ is unbalanced. 3. $\boldsymbol{\theta}$ does not modulate ${\hat{\boldsymbol{\beta}}}_{\boldsymbol{j}}$ and the distribution of $\boldsymbol{\theta}$ is balanced. 4. $\boldsymbol{\theta}$ does not modulate ${\hat{\boldsymbol{\beta}}}_{\boldsymbol{j}}$ and the distribution of $\boldsymbol{\theta}$ is unbalanced.


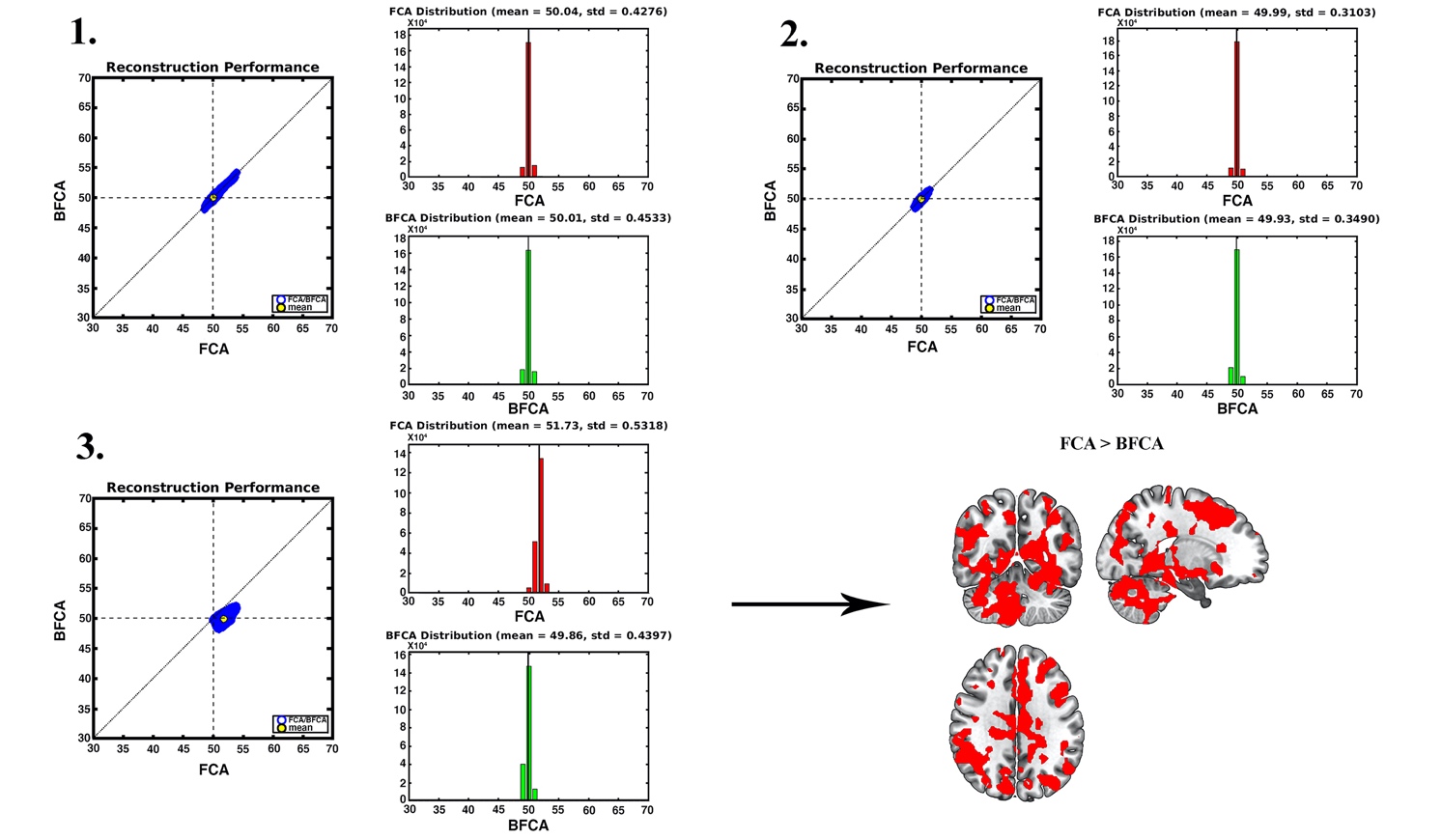


Supplementary Figure 6. The plots illustrate the difference between reconstruction performances obtained with two accuracy measures, FCA and BFCA. 1-3 show the relationship between FCA and BFCA for the 100% coherence stimulus reconstruction, for the 0% coherence stimulus reconstruction, and for the 0% coherence report reconstruction respectively. Results are plotted for each searchlight, averaged across subjects (N=23). The brain map on the bottom right is obtained from a 2^nd^ level t-test evaluating searchlights where FCA was higher than BFCA in the 0% coherence report reconstruction (N=23). The map is thresholded at p<0.001, uncorrected for multiple comparisons.
